# The Natural Product Resveratrol Inhibits Yeast Cell Separation by Extensively Modulating the Transcriptional Landscape and Reprogramming the Intracellular Metabolome

**DOI:** 10.1371/journal.pone.0150156

**Published:** 2016-03-07

**Authors:** Zhe Wang, Zhongkai Gu, Yan Shen, Yang Wang, Jing Li, Hong Lv, Keke Huo

**Affiliations:** 1 State Key Laboratory of Genetic Engineering, School of Life Sciences, Fudan University, 2005 Song-Hu Road, Shanghai, 200438, China; 2 Division of Infectious Diseases, Weill Medical College of Cornell University, 413 E 69th St, New York, NY, 10021, United States of America; 3 Institutes of Biomedical Sciences, Fudan University, 130 Dong-An Road, Shanghai, 200032, China; 4 Department of Biological Sciences and Biotechnology, State Key Laboratory of Biomembrane and Membrane Biotechnology, Tsinghua University, Beijing, 100084, China; Hiroshima University, JAPAN

## Abstract

An increasing number of studies have shown that the promising compound resveratrol treats multiple diseases, such as cancer and aging; however, the resveratrol mode-of-action (MoA) remains largely unknown. Here, by virtue of multiple omics approaches, we adopted fission yeast as a model system with the goal of dissecting the common MoA of the anti-proliferative activity of resveratrol. We found that the anti-proliferative activity of resveratrol is mainly due to its unique role of inhibiting the separation of sister cells, similar phenotype with the C2H2 zinc finger transcription factor Ace2 knock-out strain. Microarray analysis shown that resveratrol has extensive impact on the fission yeast transcription levels. Among the changed gene’s list, 40% of up-regulated genes are Core Environmental Stress Responses genes, and 57% of the down-regulated genes are periodically expressed. Moreover, resveratrol leverages the metabolome, which unbalances the intracellular pool sizes of several classes of amino acids, nucleosides, sugars and lipids, thus reflecting the remodulated metabolic networks. The complexity of the resveratrol MoA displayed in previous reports and our work demonstrates that multiple omics approaches must be applied together to obtain a complete picture of resveratrol’s anti-proliferative function.

## Introduction

Although resveratrol was first characterized in the white hellebore root in 1940 [1], this natural product has started to attract attention over the past 20 years primarily due to the discovery of its extensive pharmacological potential to treat and/or prevent diverse human diseases, such as cancer, metabolic disorders and aging-associated diseases [1–3]. Compared with the rapidly growing improvements in the animal model systems and clinical investigation, the understanding of the resveratrol’s mode-of-action (MoA), especially at the cellular and molecular levels, still remains incomplete.

Unlike many pharmaceutical compounds, resveratrol binds multiple intracellular molecular targets depending on the specific disease model [2]. For example, in terms of its anti-cancer bioactivity, resveratrol arrests the cell cycle progression of diverse cancer cell lines, which is the consequence of resveratrol binding to the RAC-α serine/threonine protein kinase (AKT) and triggering the PI3K/AKT/FOXO pathway, which is responsible for regulating the critical cell cycle controller cyclin D1 [3]. Regarding resveratrol bioactivity in extending lifespan, current evidence has demonstrated that resveratrol is the competitive inhibitor of cAMP-degrading phosphodiesterases [4]. cAMP then activates the CamKKβ-AMPK pathway through phospholipase C, increases the abundance of NAD^+^, activates Sirtuin 1 (Sirt1), and eventually improves mitochondrial function. Most recently, resveratrol was also proven to be a post-transcriptional regulator through its selective binding of the RNA-binding protein KSRP during pro-inflammatory procedures [5] and to the small RNAs miR-33a and miR-122 in hepatic cells [6].

These findings illustrate the complexity of the molecular mechanisms of resveratrol. In this research of the anti-proliferative role of resveratrol and its common MoA, we adapted a simple single cell model, *Schizosaccharomyces pombe* (*S*. *pombe*), and used multiple omics approaches to reduce the complexity and strengthen the integrity of the study. Fission yeast was chosen as the model because of the following reasons: fission yeast is a terrific model to investigate the cell cycle and cell shape; the yeast belongs to the “Crabtree Positive” yeast kingdom [7], which possesses features similar to the “Warburg Effect” of cancer cells [8] such that the fission yeast prefers to conduct fermentation with glucose as the carbon source for energy production [9]; many omics tools and datasets are available for the systematic exploration of the drug’s effects at different concentrations [10].

First, we found that resveratrol inhibits the septum degradation and delay the sister cells’ separation during the postcytokinesis period, which thus causes its anti-proliferation ability. Short-term (4 hrs) resveratrol treatment significantly decreased the expression levels of a group of cell cycle-related genes. Surprisingly, many of those impaired genes are the targets of C2H2 transcription factor Ace2 and MBF transcription complex component Cdc10. Comparative genomic analysis indicated that many of these genes are the direct downstream targets of the RFX transcription factor Sak1 and the FOXO transcription factor Fkh2, coincidentally supporting the most recently published study about Sak1 and Fkh2 working together to regulate mitotic gene expression [11]. Then, we discovered that Ace2 downstream enzymes, which control septum degradation, are related to the resveratrol-caused cell separation defects. Additionally, Ace2 is the target of its upstream factors Sak1, Fkh2 and Sep1 [11], which implies that the inhibition of the cell separation by resveratrol occurs via the modulation of these transcription factors mediated signaling pathways. Finally, gas chromatography-mass spectrometry (GC-MS)-based metabolomics analysis indicates that resveratrol has the capability of leveraging the intracellular metabolome, limiting amino acid and nucleoside biosynthesis and uptake, and switching the metabolic fluxes to produce alternative molecules, such as disaccharides and lipids. These metabolomics results clearly explained the transcriptome changed by resveratrol, such as the down-regulated gene *cdc22+* encoding a ribonucleotide reductase which directly regards to maintain the health purine pool[12]. Also, the fact that extensively down-regulated expression of transporters is consistent with the decreased multiple amino acids pool sizes. Thus, this research reveals resveratrol’s complicated MoA and demonstrates the necessity of applying multiple omics approaches at different levels to obtain the complete picture of its anti-proliferative function.

## Materials and Methods

### Yeast cell culture and drug treatment

The fission yeast *Schizosaccharomyces pombe* wild type strain 972 h^-^ was used in this research. Resveratrol was purchased from Sigma-Aldrich (St. Louis, MO, US). For the drug activity experiment, a 10 ml culture of YE medium (0.5% yeast extract, 3% glucose) was inoculated from a single colony and was grown overnight at 30°C to the late log phase (OD_600_ = 2.0–3.0). The yeast culture was then diluted to OD_600_ = 0.05 and treated with a series of resveratrol doses (0, 25, 50 100, and 200 μg/ml) in 50 ml of YE liquid culture. We measured the optical density at 600 nm at different time points (0, 4, 8, 12, 16, 20, 24, and 28 hrs), and finally the IC_50_ concentration was calculated based on the readout at 20 hrs after drug treatment.

### Cell staining, microscopic and fluorescence-activated cell sorting (FACS) analysis

Briefly, 4,6-diamidino-2-phenylindole (DAPI) nuclei staining and calcofluor septum staining were performed according to the Paul Nurse’s Lab Fission Yeast Handbook. In detail, we diluted the yeast cells from late log phase culture (OD600 = 2.0–3.0) to OD_600_ = 0.1, added the drug at its IC_50_ concentration, and collected 10^7^ cells at different time points by centrifugation at 2,500 rpm for 5 mins. Then, the cell pellets were washed once with cold ddH_2_O and were re-suspended in 1 ml of cold 70% ethanol for fixation. For DAPI and calcofluor staining, 30 μl of fixed cells were washed with 1 ml of water, and the cell pellet was re-suspended with 10 μl of water and mixed with 10 μl of 2X DAPI-calcofluorworking solution (1 μg/ml DAPI, 50 μg/ml calcofluor, 1 mg/ml p-phenylenediamine, 50% glycerol). The samples were observed under fluorescence microscopy (DM2500, Leica). For single calcofluor staining, 30 μl of fixed cells were mixed with 2X calcofluor stain (50 μg/ml calcofluor, 0.3 mg/ml p-phenylenediamine 50% glycerol). For FACS analysis, 0.3 ml of above fixed cells were washed with 50 mM sodium citrate twice. Then, RNA was firstly digested by 0.1 mg/ml RNase A at 37°C for 2 hrs and then treated with 20 mg/ml proteinase K for 1hr. Next, SYBR Green I was added to the cell suspension (1:500 dilution of the commercial solution). Just before processing the cells, a 45-sec ultrasonic treatment was applied to prevent cell conglutination. Finally, the DNA content was measured by flow cytometer using excitation at 488 nM and collecting fluorescence emission at 525 nM, and the raw data were analyzed by Flowjo software[13].

### Yeast cell size measurement

Since the unseparated cell phenotype of resveratrol treated yeast cells, the length of “whole cell chain” and the individual cell (the distances between neighbouring septa or the free cell tips and the nearest septa) were measured respectively using the software of microscopy.

### Microarray analysis and quantitative real time-polymerase chain reaction (qRT-PCR) confirmation

In total, 120 μg/ml resveratrol and vehicle control were added to YE cultured yeast at OD_600_ = 0.2. Next, the cells were incubated for 4 hrs, harvested by centrifugation, and washed once with 25 ml of cold ddH_2_O. We utilized the previously reported hot phenol method to extract the total RNA, and the RNA was purified using Qiagen RNeasy columns. The three replicated total RNA samples of each yeast sample were sent to Shanghai Biochip Co. Ltd. for Affymetrix Yeast 2.0 microarray analysis. After the standard microarray quality check and data analysis, we defined the significant gene expression changes genes (ratio>1.5, p-value<0.05). The qRT-PCR was conducted from the same RNA samples using SYBR green real-time PCR master mix (TOYOBO).

### Knock-out strain library screening

The *S*. *pombe* haploid deletion library used in this work was purchased from Bioneer (http://pombe.bioneer.co.kr/) and adapted to study the resveratrol target pathway *in vivo*. The genotypes were ED666 h+ ade6-M210 ura4-D18 leu1-32 and ED668 h+ ade6-M216 ura4-D18 leu1-32. Selected gene knock-out strains were used to perform phenotypic analysis using a microscope.

### GC-MS based metabolomic analysis

The resveratrol-treated metabolomics analysis was conducted using methyl-chloroformate derivatization followed by GC-MS. Wild-type yeast cells were treated with 120 μg/ml resveratrol treat for 4 hrs, and 1 ml of 10^8^ cell culture was mixed with 4 ml of -20°C glycerol-saline quenching solution (3:2, glycerol: 1.35% saline solution). Then, we centrifuged the quenched mixture at 36,086 g for 20 min at −20°C. The supernatant was discarded, and the cell pellet was washed with 2 ml of cold washing solution (1:1, glycerol: 1.35% saline solution). Then, 2.5 ml of cold methanol-water solution (50% (v/v), −30°C) was added to the cell pellet. Next, ribitol was added as an internal standard to each sample. The EP tubes with the mixtures were mixed vigorously using a Vortex mixer for 1 min. GC-MS analysis was performed with an Agilent 7890A gas chromatograph system coupled with the Agilent 5975C mass spectrometer. The system utilized a DB-5MS capillary column (30 mX250 μm inner diameter, 0.25 μm film thickness; J&W Scientific, Folsom, CA, USA). A 1-μl aliquot of the analyte was injected into the splitless mode. Helium was used as the carrier gas. The front inlet purge flow was 3 ml min^−1^, and the gas flow rate through the column was 1 ml min^−1^. The initial temperature was maintained at 80°C for 2 min, increased to 180°C at a rate of 10°C min^−1^, then increased to 240°C at a rate of 4°C min^−1^, and finally increased to 290°C at a rate of 25°C min^−1^ for 21 mins. The injection, transfer line, and ion source temperatures were 280°C, 270°C, and 220°C, respectively. The energy was -70 eV in electron impact mode. The mass spectrometry data were acquired in full-scan mode with the m/z range of 30 to 550 at a rate of 150 spectra per second. The acquired raw dataset was processed in SIMCA-P+ software (V11.0, Umetrics AB, Umea, Sweden) to perform the PCA and PLS-DA analysis to identify the significantly changed metabolites. The hierarchical analysis was conducted in Cluster 3.0 and visualized in Java TreeView software.

### Microarray data access

The microarray data used in this study has been deposited in Array Express Database (https://www.ebi.ac.uk/arrayexpress/) under the accession number: E-MTAB-4399.

## Results

### Resveratrol exhibits anti-proliferative activity by inhibiting sister cells’ separation

**Fig 1:**
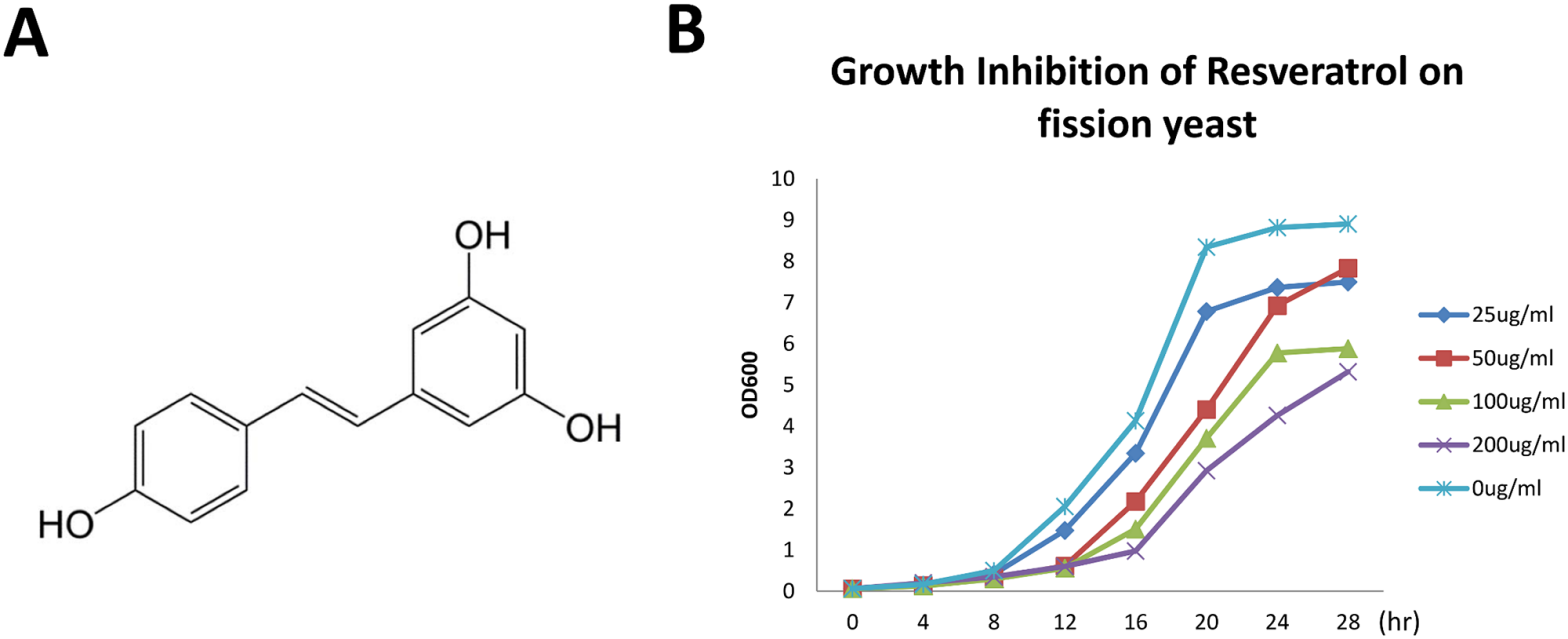
Resveratrol can inhibit the proliferation of fission yeast. (A) The structural formula of resveratrol. (B) Yeast growth inhibition curve under different doses of resveratrol (0, 25, 50, 100, and 200 μg/ml).

Earlier studies have demonstrated that the natural product resveratrol structurally belongs to the stilbenoid family (Fig 1A), a group of natural phenols frequently used in traditional medicine. Here, to explore the feasibility of using the fission yeast *S*. *pombe* as a model to study its anti-proliferative activities, we selected a series of resveratrol concentrations (0–200 μg/ml) to treat wild-type fission yeast and quantitated the anti-proliferative effect by monitoring the cell densities at OD_600_. The results showed that resveratrol inhibits cell growth in a dose-dependent kinetic manner (Fig 1B), and the IC_50_ value was 120 μg/ml. Resveratrol disrupts the cell division progress of cancer cells [14], which helps address the question-whether resveratrol-induced cell growth inhibition was related to cell division defects.

**Fig 2:**
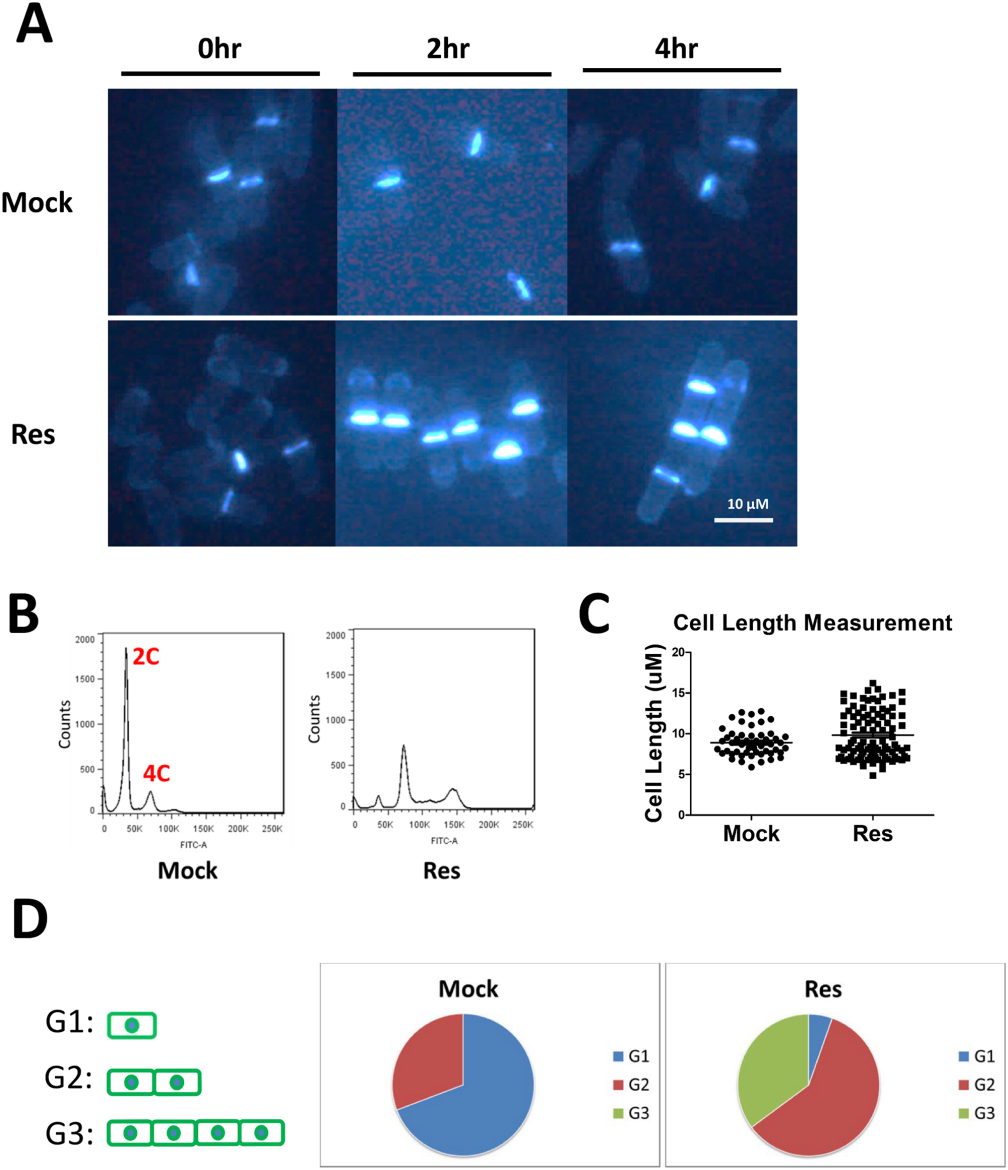
Resveratrol can inhibit cell proliferation by inhibiting sister cell’s separation. (A) Calcofluor staining to visualize the cell septum: 120 μg/ml resveratrol and mock reagent (ethanol) were used to treat the yeast cells for 0, 2 and 4 hrs (B) FACS analysis: 120 μg/ml resveratrol was used to treat the yeast cell for 0 and 4 hrs. (C) The single cell size measurement: 120 μg/ml resveratrol was used to treat the yeast cell for 6 hrs, over 50 cells were counted respectively. (D) The unsplitted septum counting: 120 μg/ml resveratrol was used to treat the yeast cell for 6 hrs, G1,G2 and G3 represent the cell chain with 0, 1 and 2 septum respectively, over 30 cells were counted.

The chemical calcofluor white has already been proven to preferentially binds onto the chitin present in the of cell septum [15]; therefore, we utilized calcofluor white as the fluorescence dye to probe the cell separation procedure. As shown in Fig 2A, after 2 hrs of treatment (at IC_50_ concentration), the septum of several cells became noticeably thicker in contrast to the mock-treated samples. The most dramatic changes occurred after 4 hrs of drug treatment, and multiple septa were observed in greater 50% of the cells, and the cell length simultaneously increased. We next conducted DAPI-calcofluor co-staining to evaluate nuclei integrity after resveratrol treatment [16]. As shown in S1 Fig, although there are clear sister cells’ separation defect in resveratrol treated cells, the sizes of individual cell didn’t change obviously (Fig 2C). The FACS analysis indicated that there is a dropped 2C-like peak and increased 4C-like peak after resveratrol treatment (Fig 2B), but which mainly due to the unbroken-up cell chain. In another word, the detected 4C amount of DNA should been the sum of the DNA contents of two or three or four non-separated cells having the normal nuclei(s). We also evaluated the severity of the resveratrol-caused sister cell’s unseparation via counting how many unsplitted septum occur in a cell chain. As indicated in Fig 2D, there are 5% cells with no septum, 56% cells with one septum and 39% cells with two septum, after 6hrs’ resveratrol treatment. On the contrary, in the mock treated group, there are 71% cells with no septum and 29% cells with one septum. In summary, resveratrol can be taken up by fission yeast and inhibit cell proliferation, most likely after the drug caused cell separation defects.

### Resveratrol regulates the cell division at a transcriptional level

**Fig 3:**
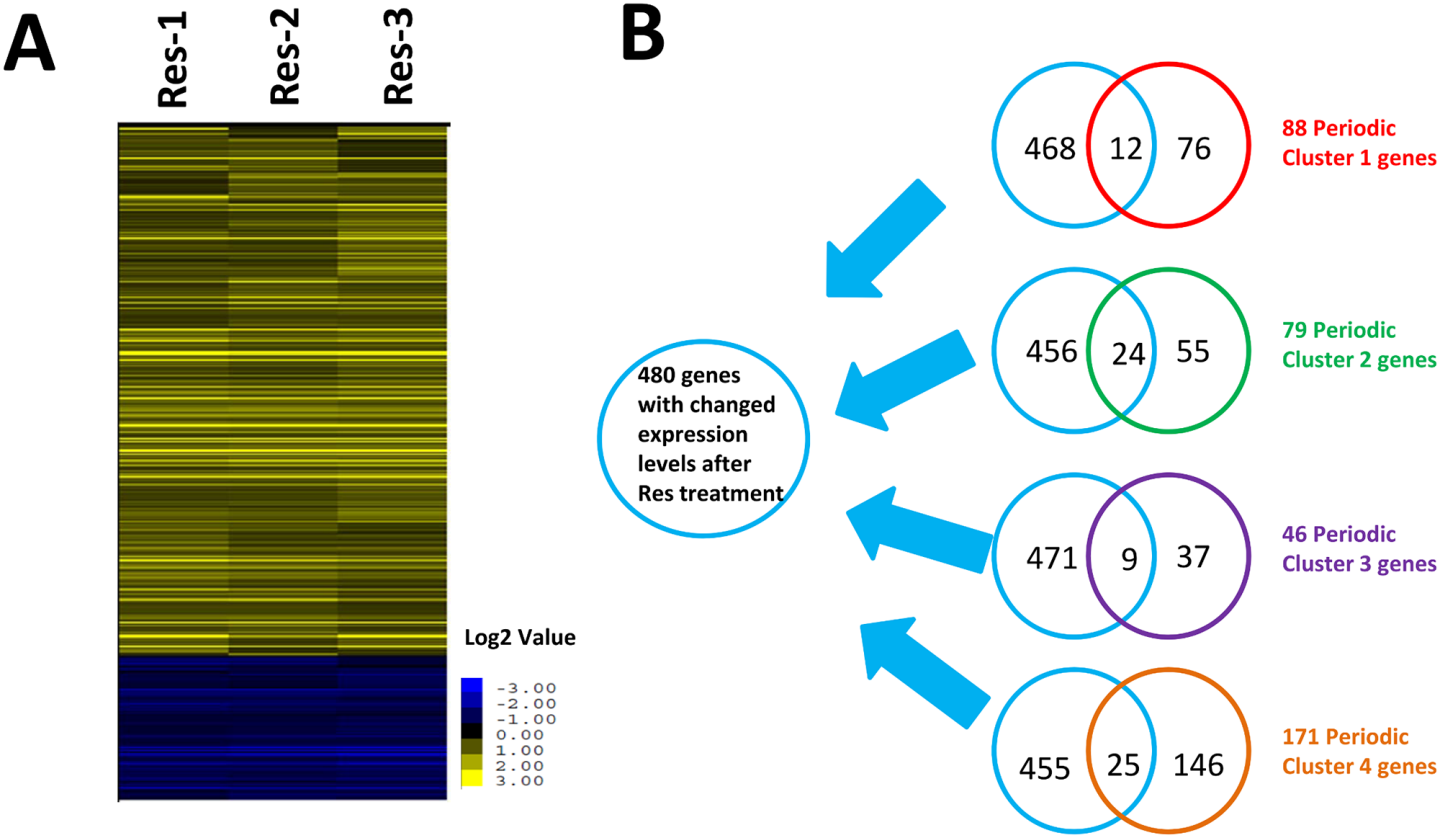
Resveratrol significantly changes the transcription levels of periodic expressed genes. (A) Clustering analysis based on the microarray analysis in which the data were selected by both the Fold Change (>50%) and p-value (<0.05). R-1, R-2, and R-3 represent three biological replicates. The blue color depicts the genes with decreased expression levels, and the yellow color depicts genes with increased expression levels. (B) The overlapping analysis between resveratrol-changed genes and previously reported periodic gene expression changes (the gene lists were adapted from Rustici et al.[19]). Cluster 1, 2, 3, and 4 with differently colors represent the gene list with different expression rhythms. In detail, the blue circle represents the 480 genes with changed expression level after resveratrol treatment; the red circle represents the previous published 88 Cluster 1 periodic genes; the green circle represents the previous published 79 Cluster 2 periodic genes; the purple circle represents the previous published 46 Cluster 3 periodic genes; the brown circle represents the previous published 171 Cluster 4 periodic genes.

With the goal of explaining this dramatic effect of resveratrol, we utilized microarray analysis to identify the gene transcriptional consequences after the drug application. By adding 120 μg/ml resveratrol to fission yeast culture at the early time point (4 hrs), we identified total 480 genes (377 gene with increased expression levels, and 103 genes with decreased expression levels) whose expression levels are significantly affected by resveratrol treatment (fold>1.5, p-value<0.05, S1 Table and Fig 3A).

We next utilized the new released web-tool AnGeLi for comprehensively analyzing the resveratrol modulated gene lists[17]. As shown in Table 1, resveratrol treatment can significantly trigger the stress related genes’ expression. For example, over 40% up-regulated genes (150 genes in total, p-value = 1.78E-71) are recognized as the “Core Environmental Stress Response Induced (CESR)” category. It’s worth nothing that previous studies have proven that the kinase Sty1 and b-ZIP transcription factor Atf1 are the key regulators of stress-dependent transcription[18]. Consistently, we found that both “Atf1 activated” and “Sty1 but not Atf1 activated genes” categories are over-represented in the statistical analysis.

**Table 1:** The gene set enchriment analysis of the resveratrol up-regulated genes.

Gene Expression	List frequency/Value	Background frequency/ Value	p-value
Core Environmental Stress Response induced	40.54% (150/370)	7.72% (541/7006)	1.79667E-71
Reproduction module	27.84% (103/370)	4.22% (296/7006)	9.01015E-57
Stress module	19.73% (73/370)	1.94% (136/7006)	2.8115E-55
Oxidative Stess Cluster 4	30.81% (114/370)	5.91% (414/7006)	1.13401E-51
Caffeine and Rapamycin induced	24.59% (91/370)	4.34% (304/7006)	1.40053E-43
Atf1 activated	12.43% (46/370)	0.97% (68/7006)	1.00503E-40
Induced usp102 mutant	9.19% (34/370)	0.64% (45/7006)	2.42075E-32
Nitrogen depletion total meiotic genes	17.57% (65/370)	3.01% (211/7006)	4.17756E-31
Meiosis sporulation module	19.19% (71/370)	3.88% (272/7006)	3.83353E-29
Nitrogen depletion continuous meiotic genes	9.73% (36/370)	0.98% (69/7006)	6.54915E-26
htb1 brl1 brl2 set1 mutant induced	12.70% (47/370)	1.84% (129/7006)	1.31747E-25
Sporulation module	14.59% (54/370)	2.68% (188/7006)	7.73829E-24
Late meiotic genes	12.70% (47/370)	2.00% (140/7006)	8.11213E-24
Induced rnc1 mutant	8.92% (33/370)	1.10% (77/7006)	3.37339E-20
Induced Amo1 mutant	5.14% (19/370)	0.54% (38/7006)	1.14912E-12
Atf31 targets	7.30% (27/370)	1.20% (84/7006)	1.40774E-12
Induced pan3 mutant	4.32% (16/370)	0.43% (30/7006)	4.28893E-11
Sty1 but not Atf1 activated genes	4.59% (17/370)	0.50% (35/7006)	5.41604E-11
Induced cig2 mutant	5.68% (21/370)	0.84% (59/7006)	1.45091E-10
H2O2 specific genes	5.41% (20/370)	0.80% (56/7006)	4.70497E-10
Induced Dbr1 deletion	14.05% (52/370)	4.92% (345/7006)	4.71908E-10
Pap1 but not Prr1 dependent genes	5.14% (19/370)	0.74% (52/7006)	1.0015E-09
Core Oxidative Stress Response	4.59% (17/370)	0.59% (41/7006)	1.18507E-09
Oxidative Stess Cluster 8	7.84% (29/370)	1.81% (127/7006)	1.75604E-09
Lowly expressed	14.59% (54/370)	5.62% (394/7006)	7.08752E-09
Stress module 2	6.76% (25/370)	1.58% (111/7006)	6.37464E-08
Induced scw1 mutant	5.41% (20/370)	1.08% (76/7006)	1.98438E-07
Ste11 targets	4.86% (18/370)	0.88% (62/7006)	2.29724E-07
Cell cycle periodically expressed	15.95% (59/370)	7.11% (498/7006)	2.9706E-07
Middle meiotic genes	16.76% (62/370)	7.88% (552/7006)	9.31455E-07
Induced SPBC56F2 08c mutant	2.16% (8/370)	0.17% (12/7006)	3.08938E-06
Induced mug187 mutant	4.32% (16/370)	0.83% (58/7006)	3.5713E-06
Induced erl1 mutant	2.70% (10/370)	0.31% (22/7006)	6.84296E-06
Oxidative Stess Cluster 1	6.76% (25/370)	2.08% (146/7006)	1.58868E-05
Induced set1 mutant	2.43% (9/370)	0.27% (19/7006)	1.90521E-05
Nitrogen depletion delayed meiotic genes	3.24% (12/370)	0.54% (38/7006)	3.19817E-05
Induced zfs1 mutant	4.32% (16/370)	0.98% (69/7006)	0.000039347
Zfs1 RNA targets	6.49% (24/370)	2.10% (147/7006)	5.81883E-05
Core Environmental Stress Response repressed	1.08% (4/370)	6.27% (439/7006)	7.14428E-05
Repressed diploid cells	5.95% (22/370)	2.37% (166/7006)	0.00376835
Caffeine and Rapamycin repressed	0.81% (3/370)	4.47% (313/7006)	0.00485944
Induced red1 mutant	3.24% (12/370)	0.90% (63/7006)	0.00564192
Early meiotic genes	4.05% (15/370)	1.40% (98/7006)	0.00964841

After analyzing the down-regulated gene list, we found there is over 57% down-regulated genes (56 genes in total, p-value = 1.19E-35) are recognized as “Cell cycle periodically expressed” category (Table 2), clearly support the cell division defect phenotype we observed after resveratrol adding. In detail, previous study has grouped the 407 “periodically expressed genes” into 4 clusters based on the rhythm and intensity of their individual expression pattern[19]. By AnGeLi analysis, we found the genes including in Cluster 2, 3 and 4 are significantly enriched in resveratrol down-regulated gene list (p-value = 3.73E-20, 2.26E-3 and 5.67E-5 respectively, shown as Table 2 and Fig 3B), suggesting that resveratrol modulated cell division by transcriptional reprogramming. It is worth noting that many above hits are actually the targets of two transcription factors, Ace2 and Cdc10, suggests the critical role of transcription factors in the MoA of resveratrol. Another interesting finding is that many genes encoding transporters are also down-regulated by resveratrol treatment. Functionally they were annotated as anion transmembrane transporters, amino acid transporters, carboxylic acid transporters, organic acid transporter, sulfur compound transporters et al, which implies that nutrients uptake capabilities could be remodulated by resveratrol.

**Table 2:** The gene set enchriment analysis of the resveratrol down-regulated genes.

Gene Expression	List frequency / Value	Background frequency / Value	p-value
Cell cycle periodically expressed	57.14% (56/98)	7.11% (498/7006)	1.1904E-35
Best periodic genes	30.61% (30/98)	1.93% (135/7006)	2.29709E-25
Cell Cycle Cluster 2	23.47% (23/98)	1.30% (91/7006)	3.73359E-20
Ace2 targets	12.24% (12/98)	0.36% (25/7006)	2.40086E-13
Cadmium specific	11.22% (11/98)	0.46% (32/7006)	3.49312E-10
Nitrogen depletion total meiotic genes	20.41% (20/98)	3.01% (211/7006)	4.60556E-09
Caffeine and Rapamycin induced	23.47% (23/98)	4.34% (304/7006)	9.81243E-09
Cdc10 targets	8.16% (8/98)	0.34% (24/7006)	2.23714E-07
Reproduction module	20.41% (20/98)	4.22% (296/7006)	9.13303E-07
htb1 brl1 brl2 set1 mutant repressed	14.29% (14/98)	1.97% (138/7006)	0.000001427
Repressed usp102 mutant	7.14% (7/98)	0.44% (31/7006)	0.000037843
Cell Cycle Cluster 4	13.27% (13/98)	2.27% (159/7006)	5.67132E-05
Repressed Dbr1 deletion	15.31% (15/98)	3.33% (233/7006)	0.000128027
Repressed spt6 mutant	6.12% (6/98)	0.39% (27/7006)	0.000254072
Induced diploid cells	9.18% (9/98)	1.38% (97/7006)	0.00113967
Induced mug187 mutant	7.14% (7/98)	0.83% (58/7006)	0.00193038
Repressed gar2 mutant	7.14% (7/98)	0.84% (59/7006)	0.00212742
Cell Cycle Cluster 3	7.14% (7/98)	0.86% (60/7006)	0.00226204
anion transmembrane transport	13.2 (13/98)	1.3 (96/7006)	0.000000186
anion transport	14.2 (14/98)	1.6 (119/7006)	0.000000238
amino acid transport	8.1 (8/98)	0.6 (43/7006)	0.000026
carboxylic acid transport	9.1 (9/98)	0.8 (60/7006)	0.0000274
organic acid transport	9.1 (9/98)	0.8 (61/7006)	0.0000308
sulfur compound transport	6.1 (6/98)	0.2 (21/7006)	0.0000617
organic anion transport	10.2 (10/98)	1.2 (88/7006)	0.0000674

### Genetic knock-out screening facilitates the identification of the drug targeted pathways

**Fig 4:**
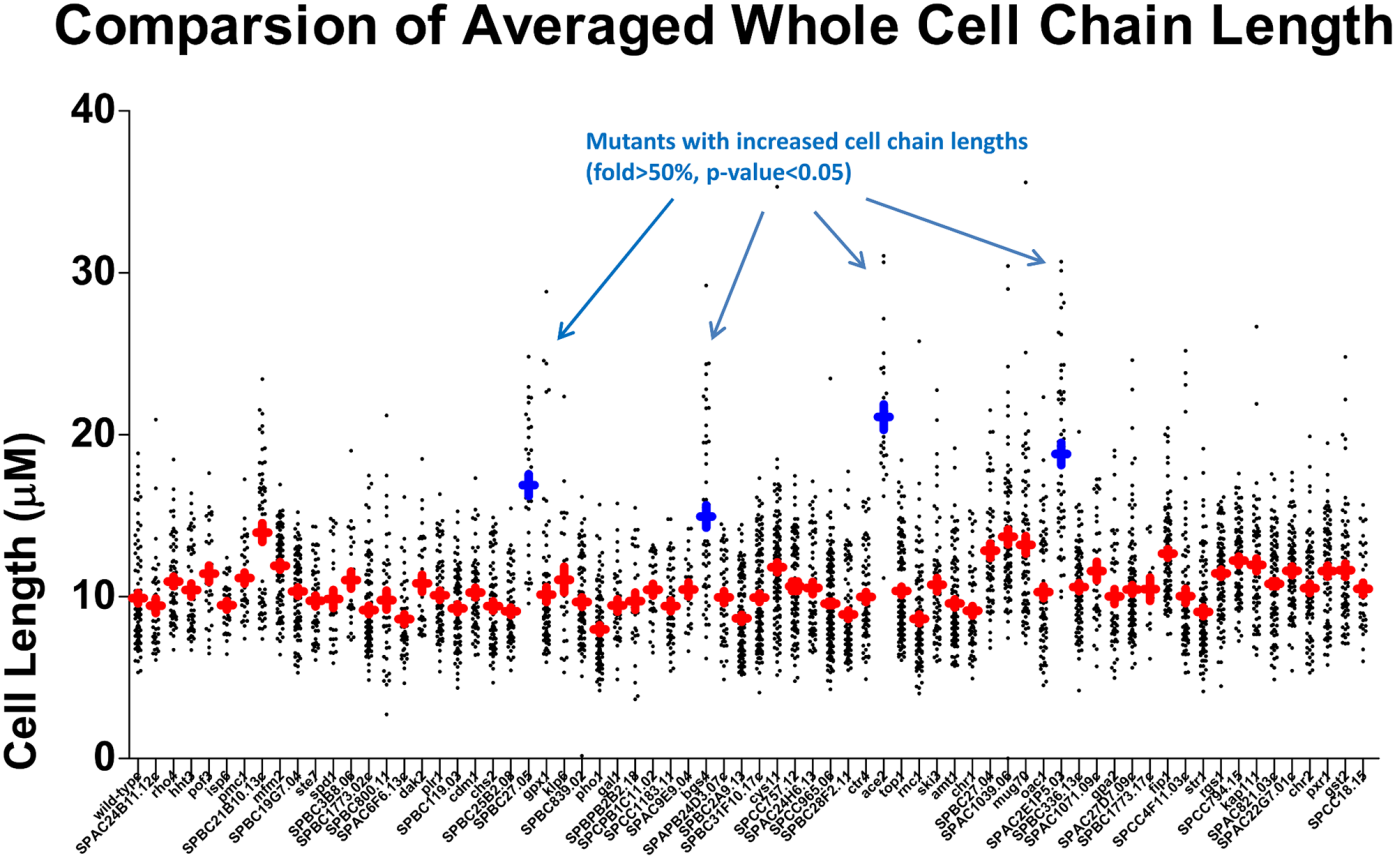
Averaged “whole cell chain” lengths of candidate knock-out strains. The “whole cell length chain” distribution of all the selected 69 candidate knock-out strains. The red points represent the median value of the cell lengths, and the blue points represent the mutants with the significantly increased whole cell chain lengths (fold>1.5, p-value<0.05). The gene name of systemic ID of these strains listed in this figure are (from left to right): wild-type, SPAC24B11.12c, rho4, hht3,pof3, isp6, pmc1, SPBC21B10.13c, mfm2, SPBC19G7.04, ste7, spd1, SPBC3B8.06, SPBC1773.02c, SPBC800.11, SPAC6F6.13c, dak2, plr1, SPBC119.03, cdm1, chs2, SPBC25B2.08, SPBC27.05, gpx1, klp6, SPBC839.02, pho1, gal1, SPBPB2B2.18, SPCPB1C11.02, SPCC1183.11, SPAC9E9.04, bgs4, SPAPB24D3.07c, SPBC2A9.13, SPBC31F10.17c, cys11, SPCC757.12, SPAC24H6.13, SPCC965.06, SPBC28F2.11, ctr4, ace2, top1, rnc1, ski3, amt1, chr1, SPBC27.04, SPAC1039.06, mug70, oac1, SPAC2E1P5.03, SPBC336.13c, SPAC1071.09c, gpa2, SPAC27D7.09c, SPBC1773.17c, fip1, SPCC4F11.03c, str1, rgs1, SPCC794.15, kap111, SPAC821.03c, SPAC22G7.01c, chr2, pxr1, gst2, SPCC18.15.

Combining the phenotypic measurement with transcriptome analysis, we hypothesized that the inhibition of fission yeast cell division caused by resveratrol is at least partially dependent on transcriptional reprogramming. As shown by the microarray analysis, resveratrol has the capability of down-regulating the expression of a group of cell cycle-related genes; therefore, we hypothesized that this decreased abundance of mRNAs would contribute to resveratrol-mediated phenotypic changes. From the whole gene list that down-regulated after resveratrol treatment, we found 69 of them are nonessential genes and we can acquire from the *S*. *pombe* haploid deletion library. We therefore cultured these 69 knock-out mutant strains and measured the sizes of their entire cell chain to look for the similar phenotype with resveratrol treated cells. As shown in Fig 4, of the total 69 mutant strains, there are 4 mutants with the obvious increased size of cell chain (fold>1.5, p-value<0.05). Among them, the *ace2Δ* strain exhibited most serious cell division arrest, with almost doubled average cell chain length. Among other three candidates, SPBC27.05 don’t has clear annotation information; Bgs4 is a subunit of 1,3-beta-glucan synthase, which is annotated to take part in the primary cell septum biogenesis[20]; SPAC2E1P5.03 is predicted as a DNAJ domain protein, which is known that has negative genetic interaction with MBF transcription factor complex (Cdc10 is the subunit of it)[21]. These results confirmed the important functional role of transcription factors and downstream septum splitting related enzymes in the MoA of resveratrol.

### Resveratrol treatment caused similar phenotypes and molecular signatures as knocking out *ace2+* gene

**Fig 5:**
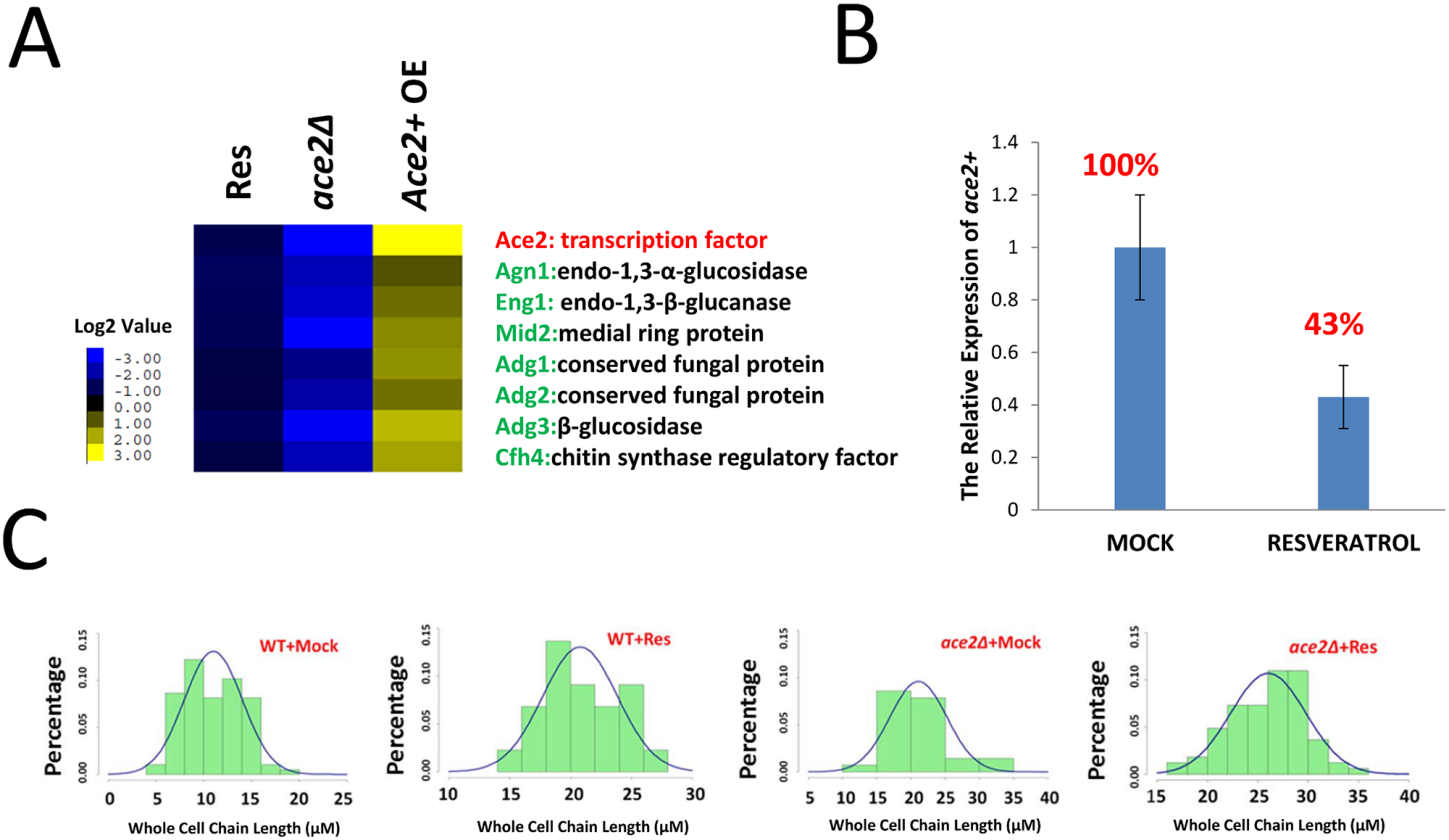
Resveratrol causes the similar cell phenotype and molecular signatures as knocking out *ace2+ in vivo*. (A) Clustering analysis of resveratrol-induced genes and previously reported Ace2 overexpression and knock-out datasets. Res denotes the resveratrol-treated sample, *ace2Δ* denoted the *ace2+* gene knock-out sample, and *ace2+* OE denoted the *ace2+* gene overexpression sample. The *ace2Δ* and *ace2+* OE datasets were downloaded from the website of Prof. Jürg Bähler from UCL. (B) The relative gene expression level of *ace2+* after resveratrol treatment (120 μg/ml resveratrol treat 4 hrs). (C) The whole cell chain length measurement and statistical distribution of wild-type and *ace2Δ* yeasts with or without resveratrol treatment counted in more than 30 cells for every yeast sample.

Above microarray and genetic phenotype analysis suggests an important role of *ace2+* with regard to the MoA of resveratrol. The microarray result of *ace2+* is first confirmed by real-time qPCR (Fig 5B). As a well-studied gene in fission yeast, *ace2+* encodes a C2H2 zinc finger transcription factor, and a previous study has shown that Ace2 controls the expression levels of several groups of downstream genes [22]. Among them, Group 1 genes include endo-α-1, 3-glucanase (Agn1) and endo-β-1, 3-glucanase (Eng1). During cytokinesis, the final step is the dissolution of the primary septum, which is primarily catalyzed by Agn1 and Eng1 [23]. An unessential gene, *mid2+*, which belongs to Group 2, exhibits a chained phenotype, which is caused by an abnormal septin ring [24]. Group 3 genes include *Adg1*, *Adg2*, *Adg3* and *Cfh4* [24], where "Adg" means "Ace2-dependent genes". Among them, both Adg1 and Adg2 are Ser/Thr-rich proteins; however, their biological functions remain unclear. Adg3 is a serine-rich protein, which has been predicted to have glucosidase activity. All of the three *adg+* genes have several “CCAGCC” motifs located in their promoter regions, and this motif is a known Ace2 intracellular binding site. Cfh4 is annotated as a regulatory subunit of chitin synthase III, but its real function still remain unclear since there lacks chitin in fission yeast cell wall. All of these genes are hypothesized to be involved in the mother/daughter cell separation processes. In this study, two lines of evidence support the hypothesis that resveratrol targeted the Ace2 transcription factor-mediated signaling pathways *in vivo*. First, when clustering the resveratrol-caused expression profiling with earlier published *ace2+* knock-out and overexpression datasets [22], we found that both *ace2+* and its downstream target genes were down-regulated by the resveratrol treatment (Fig 5A). Second, compare to the almost 100% increased whole cell chain length of resveratrol treated wild-type cells, the whole cell chain length of drug treated *ace2Δ* cell are increased about 35% (20 μM to 27 μM, p-value<0.05, Fig 5C), suggests although the down-regulation of *ace2+* is critical to the resveratrol caused phenotype, there are other factors, also contributes the MoA of resveratrol.

A recent study shown that fission yeast transcription factor Sak1, Fkh2, and Sep1 are critical for controlling the cell mitosis. After comparatively analyzing the Chip-seq datasets of transcription factors Sak1, Fkh2 and Sep1 [11], many of the downstream binding targets of these transcription factors were also significantly regulated by resveratrol (11 genes/20% totally for Sak1, 32 genes/22% totally for Fkh2, 4 genes/44% totally for Sep1, S2 Table). Interestingly, *ace2+* is just co-regulated by Sak1, Fkh2, and Sep1. These results demonstrate the importance of transcription factor Ace2 mediated signaling pathway in the resveratrol’s MoA.

### Widespread modulation of metabolic activity by resveratrol treatment

Earlier studies have explored resveratrol function in metabolic regulation. Park et al. showed that resveratrol directly targets and then inhibits the cAMP-dependent phosphodiesterases [4], increasing the intracellular cAMP concentration. Therefore, resveratrol triggers the AMPK cascade and causes multiple physiological outputs, including the down-regulation of reactive oxygen species (ROS) and glycolysis, and the up-regulation of mitochondrial respiration and gluconeogenesis. Another study assessed the effects of resveratrol on a breast cancer cell line via the liquid chromatography-mass spectrometry (LC-MS) technique, and the result revealed the significant impact of resveratrol on several amino acids or biogenic amines [25]. In this study, to explore the relationship between metabolite remodeling and cell cycle reprogramming and to integrate the information regarding the drug effects at transcriptional and metabolic levels, we adopted GC-MS-based metabolomics to measure the pool size of a list of metabolites.

**Fig 6:**
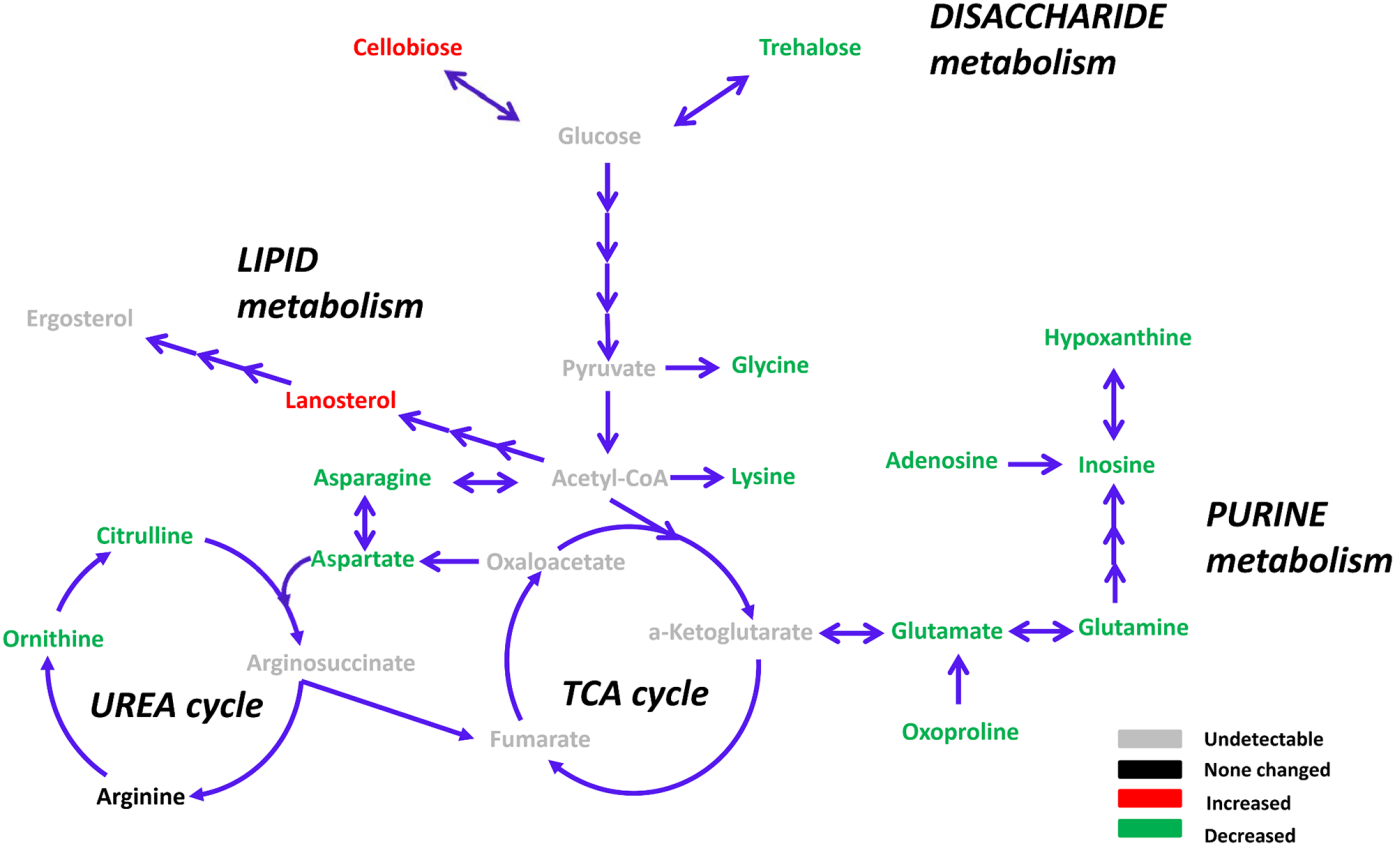
Resveratrol Modulates the Cell Metabolome. Intracellular pool sizes of metabolic intermediates in the TCA cycle, urea cycle, glycolysis, lipid metabolism pathway, disaccharide metabolism pathway and purine metabolism pathway. Grey fonts represent the undetectable metabolites. The black font represents the unchanged metabolites. The red font represents the significantly increased metabolites (increased pool size>50%, p-value<0.05), and the green font represents the significantly decreased metabolites (decreased pool size>50%, p-value<0.05). The quantitated pool size changes are indicated in Table 3.

After the targeted-metabolomics analysis, we identified a list of metabolites with markedly changed intracellular pool sizes (fold>1.5, and p-value<0.05, shown in Fig 6 and Table 3), most of which were involved in cellular energy metabolism (Table 4). Among them, we found that the concentrations of multiple amino acids were down-regulated after resveratrol treatment, including lysine (49% left), asparagine (46% left), glutamate (42% left), oxoproline (38% left), glycine (35% left), ornithine (26% left), citrulline (28% left), aspartate (22% left), and glutamine (15% left). Multiple amino acid depletion is the clear hallmark of a decrease in translational activity [26] due to the decrease in the intracellular substrate concentrations necessary for the protein biosynthesis machinery. Additionally, both aspartate and ornithine are components in the urea cycle, which converts toxic ammonia to non-toxic urea [27]. The urea cycle consumes 4 ATP molecules and produces two NADH molecules; thus, the urea cycle slightly releases more energy than it consumes [27]. Therefore, we hypothesized that resveratrol also limits the energy generation process, coordinating with the decreased cell metabolic requirement. Another group of depleted metabolites included intermediates in the purine metabolism pathway. We found that the inosine (35% left), adenosine (38% left), xanthine (40% left) and hypoxanthine (34% left) levels decreased dramatically. This phenotype was consistent with the previously reported observation that resveratrol effectively inhibits ribonucleotide reductase [28] and delays *de novo* DNA synthesis, whose significant up-regulation is a known feature in rapidly proliferating cancer cells [29]. Moreover, among these down-regulated metabolites, we found that the intracellular concentrations of two disaccharides (gentiobiose and cellobiose) were significantly increased by 211% and 184%, respectively, which may be the metabolic signature of delayed cell wall hydrolysis. We also observed that the pool size of one steroid-lanosterol was increased by 79%. These results suggest that resveratrol converts energy to the form of sugars and lipids, instead of simply damaging cells and leaking all nutrients.

**Table 3:** The Changed Metabolome After Resveratrol Treatment.

ID of Metabolits	RT (min)	p-value	Fold Change
Lipoic acid	30.947	0.003	6.861
Cellobiose	30.486	0.002	3.114
Carnitine	28.502	0.030	3.055
Gentiobiose	30.213	0.001	2.845
Adipamide	22.607	0.040	2.747
Lanosterol	37.968	0.043	1.786
Uracil	9.491	0.001	0.497
Lysine	17.908	0.001	0.494
Asparagine	11.647	0.001	0.462
Nicotinamide	11.438	0.000	0.461
Glutamic acid	13.122	0.004	0.425
Xanthine	19.642	0.002	0.399
Oxoproline	11.881	0.004	0.379
Adenosine	29.445	0.000	0.375
Inosine	28.966	0.000	0.374
Glycine	6.406	0.018	0.351
Inosine	28.978	0.000	0.349
Hypoxanthine	15.904	0.000	0.337
Galactinol	17.127	0.001	0.331
Trehalose	30.297	0.000	0.317
Nicotinic acid	8.925	0.006	0.314
Citrulline	16.213	0.000	0.278
Ornithine	13.057	0.002	0.259
Creatine	29.748	0.006	0.243
Aspartic acid	11.843	0.001	0.223
Glutamine	11.266	0.016	0.146
Myo-inositol	21.016	0.009	0.121

The changed metabolites identified by both p-values (<0.05) and fold changes (>50%).

**Table 4:** The Pathway Analysis Based on the Changed Metabolome.

Pathway Name	Total	Hits	p-value	-log(p)
Purine metabolism	1.1849	5	0.005651	5.1759
Starch and sucrose metabolism	0.64396	3	0.025196	3.6811
Alanine, aspartate and glutamate metabolism	0.3091	2	0.037161	3.2925

The statistically enriched pathways based on the changed metabolome as conducted in the online software MetaboAnalyst 2.0[30].

## Discussion

Despite the widely recognized pharmacological potential of the plant-derived compound resveratrol, its underlying MoA remains incompletely defined. Our studies elucidated that the transcription factor-mediated cell division inhibiting and global metabolic reprogramming are the major MoA of resveratrol in response to its anti-proliferative function in fission yeast. We specifically identified that resveratrol can extensively modulate the transcriptional landscape, by virtue of impacting on the gene expression of multiple cell division related transcription factors and nutrients transporters. The changed transcriptome next reprograms the intracellular metabolome, immediately switches the metabolic fluxes from the mode of supporting fast cell division to the one of controlling energy production with increased energy store.

**Fig 7:**
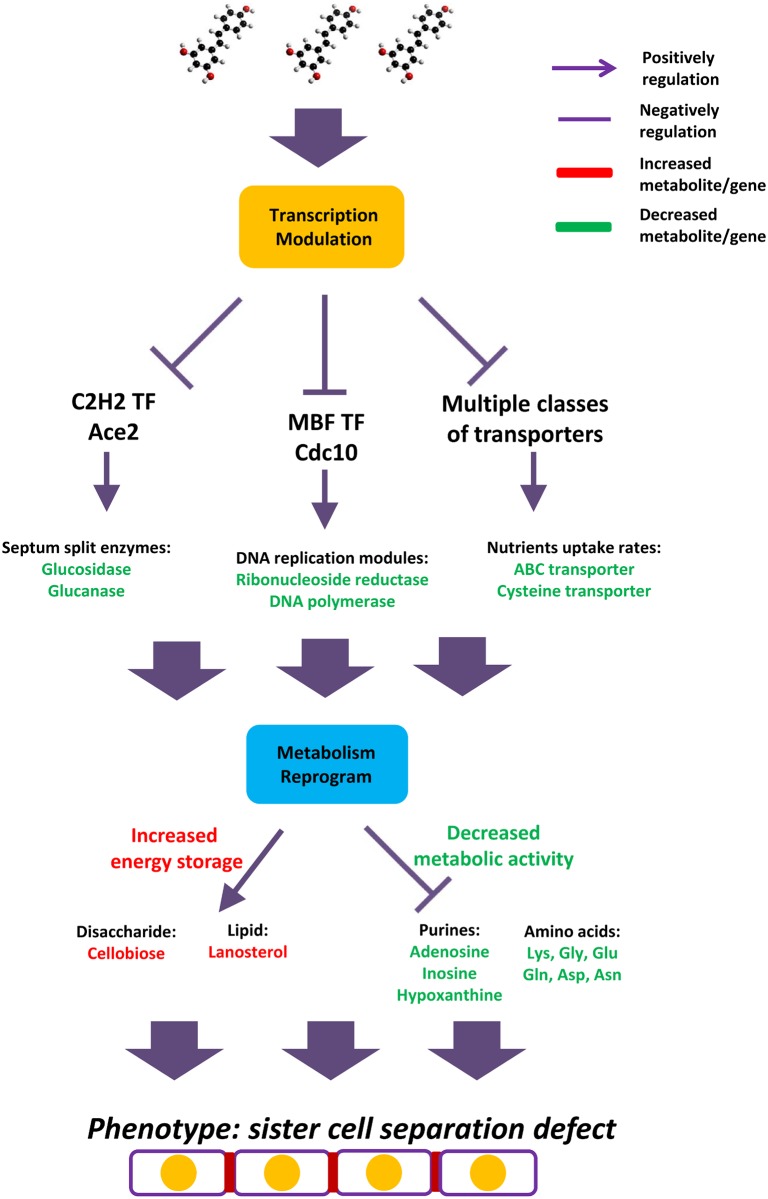
The Mode-of-action of Resveratrol’s Anti-proliferative Activity in Fission Yeast. As shown in this figure, once resveratrol penetrates the cell wall/membrane, this compound will first extensively change the transcriptional landscape via the transcription factor Ace2, Cdc10 and multiple transporter genes. As the downstream consequences of down-regulated Ace2, septum hydrolysis enzymes, such as glucosidase, glucanase and septum ring-related protein are down-regulated at the transcription level, which causes the delay of septum splitting. As the target gene of Cdc10, the nucleoside reductase and possibly DNA replication is impaired, then causes the decreasing of intracellular purines, such as adenosine, inosine and hypoxanthine. As the result of down-regulated multiple transporter genes, the nutrient uptake is impaired, thus triggers the widely shrinking amino acid pool size. The cell also up-regulates the abundances of cellobiose and lanosterol, which is supposed to improve the intracellular energy store.

Multiple studies have reported that resveratrol has the strong capability to arrest the cell cycle and inhibit cancer cell growth. For example, resveratrol can arrest the breast cancer cell line MDA-MB231 [31], the hepatocellular carcinoma cell line Hep3B [32], the colon cancer cell line HT-29 [33], and the lung adenocarcinoma cell line A549 [34]. Moreover, the eukaryotic cell cycle procedure is tightly controlled at the gene expression level [35], and fission yeast has been proven to be the perfect model to investigate these procedures [36] due to its multiple advantages. Based on the fission yeast model, Rustici et al. reported on the genome-wide transcriptional program of fission yeast cell cycle and characterized a total of 407 periodically expressed genes, which are individually regulated by several transcription factors that subsequently induce respective expression waves [19]. Oliva et al. also reported that 750 genes have a significant oscillation expression pattern, most possibly due to the regulation by specific transcription factor(s), which is supported by the evidence that conserved DNA sequence motifs are enriched in their gene promoter regions [37]. Not surprisingly, earlier studies have proven that transcription factor-mediated gene expression regulation is part of the resveratrol MoA. For example, in prostate cancer cells, resveratrol has been reported to inhibit the phosphorylation of the forkhead (FOXO) transcription factor and results in its nuclear translocation, DNA binding and transcriptional activity [38]. In fission yeast, the FOXO transcription factors Fkh2 and Sep1 regulate the periodic expression of *cdc15+* and *spo12+* and are critical to maintaining normal cell cycle processes [39]. Most recently, Garg et al. reported that the RFX family transcription factor Sak1 works with the forkhead factors Fkh2 and Sep1 to regulate mitotic expression in fission yeast [11]. Surprisingly, when comparatively analyzing the chip-seq datasets with our resveratrol-regulated gene datasets, we found that the high proportions of Sak1, Fkh2 and Sep1 downstream target genes are also changed by resveratrol treatment at the transcription level. Interestingly, a C2H2 transcription factor Ace2 is co-regulated by the Sak1, Fkh2 and Sep1. Based on our results, the gene expression level of *ace2+* is down-regulated by resveratrol, and the *ace2+* null mutants shares a similar phenotype with the drug treated wild-type yeast, including elongated cell length and sister cell separation defects. Earlier studies related to Ace2 have demonstrated that this protein binds to the “CCAGCC” motif and specifically regulates the transcription of multiple enzymes related to septum splitting, such as endo-α-1,3-glucanase (Agn1), endo-β-1,3-glucanase (Eng1), and other cell separation-related genes [32]. Our study extends this finding with the discovery of a unique “Sak1/Fkh2/Sep1---Ace2---enzyme” cascade that contributes to resveratrol-mediated sister cells’ separation and anti-proliferative activities (Fig 7).

From the gene set enrichment analysis, we also found that the target genes of *cdc10+* are highly representative in the down-regulated gene list. It is well known that *cdc10+* is the critical component of MBF transcription complex and takes part in the gene regulation during cell cycle[40]. In this research, we saw that at least 8 *cdc10+* target genes had the decreased expression level after resveratrol treatment. Among them, *cdc22+* (ribonucleotide reductase large subunit, [12]) and *dut1+* (deoxyuridine 5'-triphosphate nucleotidohydrolase, [41]) are critical enzymes for the metabolism of nucleosides and nucleotides, which therefore causes the following dropped pool sizes of the several metabolites in purine metabolism pathway. Other *cdc10+* targets includes several genes directly related to DNA replication process, such as *cdt1+* (replication licensing factor, [42]), *mik1+* (mitotic inhibitor kinase, [43]), and *pol1+* (DNA polymerase alpha catalytic subunit, [44]). Above observation addresses the possibility that resveratrol impacts on DNA replication process, but the more detailed mechanism remains to be elucidated.

The decreased expression levels of several classes of transporter genes are the resveratrol-caused transcriptional signatures as well. For example, the gene list includes the amino acid transporter (SPBPB10D8.01, [21]), biotin transporter (*vht1+*, [21]), ABC transporter (*abc3+*, [45]), sulfate transporter (SPAC24H6.11c, [21]) et al. Above results in gene transcription level clearly suggest a decreased capacities of nutrient uptake and impaired metabolic activity. Above hypothesis is next confirmed by the experimental result that resveratrol can down-regulate the intracellular pool sizes of a group of amino acids, includes lysine, asparagine, glutamate, oxoproline, glycine, ornithine, citrulline, aspartate, and glutamine. Therefore our studies also identified resveratrol’s unique role in metabolic modulation. The widespread decreased intracellular amino acid pool sizes have been recognized as the predictors of slowing entire protein synthesis rates because the availabilities of these amino acids are critical for fueling the translational machinery [46]. Moreover, the biosynthesis of amino acids is tightly associated with central carbon metabolism (CCM) [47]. For example, the TCA cycle intermediate α-ketoglutarate is the direct precursor for synthesizing glutamate and glutamine [48], and oxaloacetic acid is the precursor for synthesizing aspartate [47]. Therefore, we predicted the decline in multiple amino acids levels, possibly followed by decreased translational efficiency, was the consequences of resveratrol-mediated restriction of cellular energy production.

Simultaneously, the increased abundance of intracellular steroid and cellobiose indicated that the cellular metabolome was leveraged by resveratrol; therefore, the metabolic fluxes were switched from the mode of supporting fast cell division to the one of controlling energy production with increased energy storage (Fig 7). In general, the observed metabolic changes shaped the unique metabolic signature of resveratrol.

In summary, in this study, we elucidated that the anti-proliferative compound resveratrol inhibits sister cells’ separation, primarily through the transcription factor Ace2 and Cdc10-controlled signaling pathways and triggers the extensive metabolic reprogramming for facilitating the slow growing requirement. Moreover, the complexity of the resveratrol MoA demonstrates that it is essential to apply multiple omics approaches to obtain a complete picture of its anti-proliferative function.

## Supporting Information

S1 Fig The nuclei and septum co-staining shows resveratrol mainly effect on sister cell splitting step. (A) The untreated control group. (B) The resveratrol treated 6hr’s group.
(TIF)

S1 Table The gene lists that have obviously changed expression levels after resveratrol treatment. The gene lists that include the resveratrol-caused up-regulated and down-regulated genes, which are identified by the criteria of both p-value (<0.05), and fold changes (>50%). (A) The group with increased gene expression level. (B) The group with decreased gene expression level.
(PDF)

S2 Table Resveratrol Regulated Genes are Direct Target of Sak1/Fkh2/Sep1 Transcription Factors. The gene lists indicates the overlap between resveratrol regulated genes and Sak1/Fkh2/Sep1 transcription factor downstream binding target genes (the Chip-seq datasets were adapted from Garg A et al. [11]).
(PDF)
