# Supplementary figures and images for: The Natural Product Resveratrol Inhibits Yeast Cell Separation by Extensively Modulating the Transcriptional Landscape and Reprogramming the Intracellular Metabolome
Source: PLoS One. 2016 Mar 7;11(3):e0150156. doi: 10.1371/journal.pone.0150156 (PMC4780762; doi:10.1371/journal.pone.0150156)

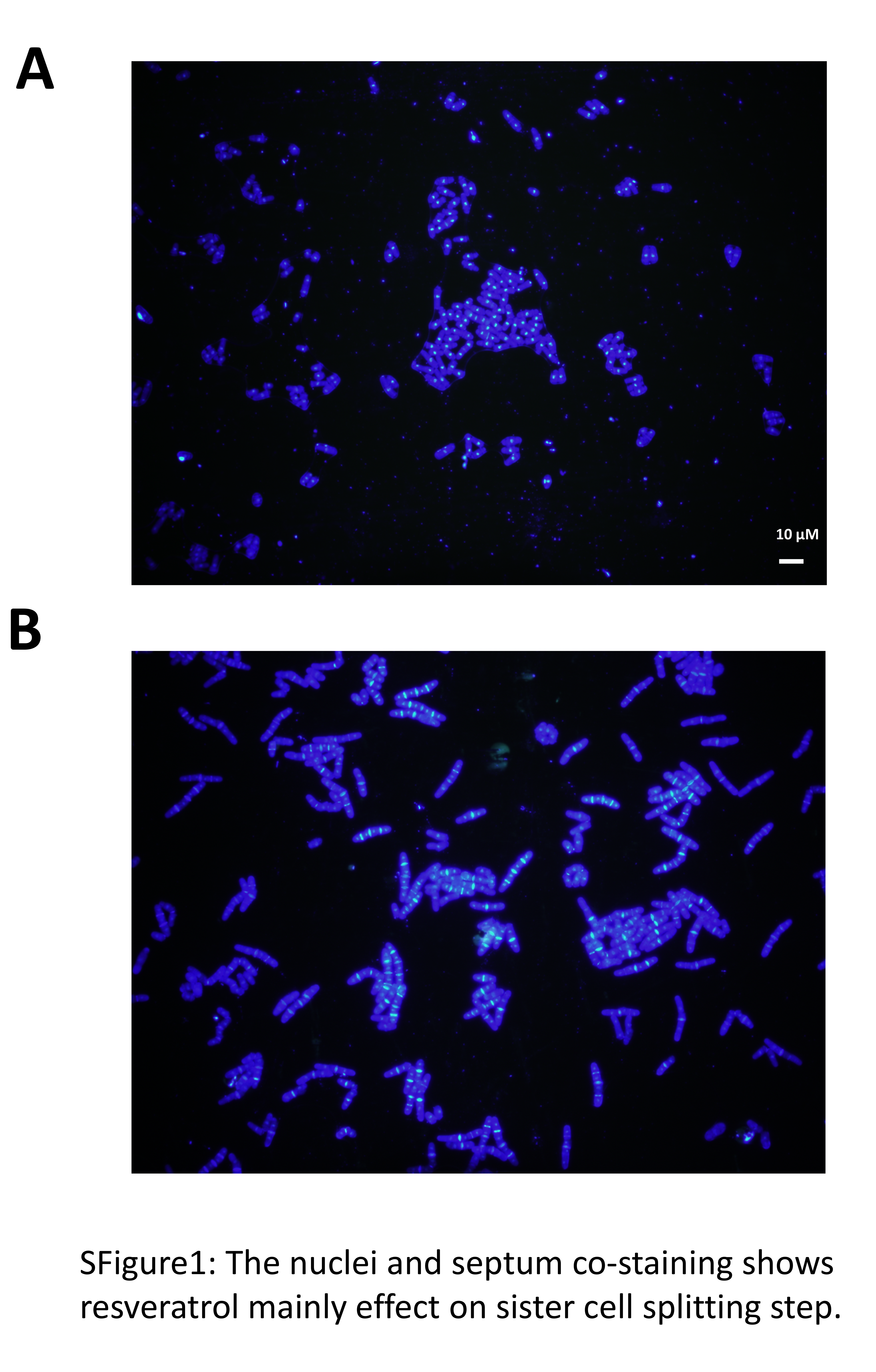

Supplement: S1 Fig — (A) The untreated control group. (B) The resveratrol treated 6hr’s group. (TIF) [file pone.0150156.s001.tif]
