# Supplementary material for: The Natural Product Resveratrol Inhibits Yeast Cell Separation by Extensively Modulating the Transcriptional Landscape and Reprogramming the Intracellular Metabolome
Source: PLoS One. 2016 Mar 7;11(3):e0150156. doi: 10.1371/journal.pone.0150156 (PMC4780762; doi:10.1371/journal.pone.0150156)
Supplement: S1 Table — The gene lists that include the resveratrol-caused up-regulated and down-regulated genes, which are identified by the criteria of both p-value (<0.05), and fold changes (>50%). (A) The group with increased gene expression level. (B) The group with decreased gene expression level. (PDF) [file pone.0150156.s002.pdf]

Supplemental Table 1A: The gene list that up-regulated by resveratrol

Transcript_ID(Array_Design)	Gene_Symbol	Relative Ratio	p-value	Regulation	Gene_Title
SPBC11C11.06c	SPBC11C11.06c	1.6412555	0.000332	up	sequence orphan
SPCC1235.01	SPCC1235.01	2.025085	0.024478117	up	sequence orphan
SPCC1183.09c	pmp31	2.2080731	0.0000269	up	plasma membrane proteolipid Pmp31
SPBP22H7.03	SPBP22H7.03	1.5109093	0.000973	up	sequence orphan
SPAC1039.07c	SPAC1039.07c	1.5460242	0.001819365	up	2,2-dialkylglycine decarboxylase (predicted)
SPBC215.15	sec13	1.5679578	0.014772256	up	COPII-coated vesicle component Sec13
SPAC17A5.10	SPAC17A5.10	1.8490736	0.000279	up	conserved fungal protein
SPBC3H7.06c	pof9	1.7932706	0.021264687	up	F-box protein Paf9
SPBC19C7.05	SPBC19C7.05	5.4628763	0.008687849	up	cell wall organization protein (predicted)
SPAC1142.09	SPAC1142.09	1.9411763	0.042283	up	dubious
SPBP4H10.12	SPBP4H10.12	1.6712093	0.00032	up	conserved protein (fungal and bacterial)
SPAC1687.14c	SPAC1687.14c	1.6083881	0.02734501	up	EF hand family protein, unknown role
SPBC119.07	ppk19	1.5323517	0.01233171	up	serine/threonine protein kinase Ppk19
SPAC22H10.13	zym1	4.8547597	0.00153772	up	metallothionein (PMID 12050156)
SPAC32A11.02c	SPAC32A11.02c	2.278425	0.0000419	up	conserved fungal protein
SPBC1652.01	SPBC1652.01	1.7102374	0.000146	up	conserved fungal protein
SPBC24C6.09c	SPBC24C6.09c	6.246148	0.000171	up	phosphoketolase (predicted)
SPAC20G4.03c	hri1	3.6443756	0.000232	up	eIF2 alpha kinase Hri1
SPCC320.14	SPCC320.14	2.2482839	0.007310192	up	threo-3-hydroxyaspartate ammonia-lyase (predicted)
SPAC186.02c	SPAC186.02c	3.4238863	0.009831117	up	hydroxyacid dehydrogenase (predicted)
SPAC8C9.16c	SPAC8C9.16c	1.657457	0.020681217	up	TLDc domain protein 1
SPCC1183.11	SPCC1183.11	1.8010888	0.011138435	up	MS ion channel protein 1
SPAC4H3.04c	SPAC4H3.04c	2.1021583	0.0000531	up	UPF0103 family
SPCC1020.05	SPCC1020.05	1.523514	0.047306284	up	phosphoprotein phosphatase (predicted)
SPBC14C8.05c	meu17	1.6946914	0.001229521	up	glucan-alpha-1,4-glucosidase
SPCP1E11.05c	SPCP1E11.05c	1.9566991	0.015090602	up	sterol O-acyltransferase (predicted)
SPBP35G2.13c	swc2	1.5683713	0.001130118	up	chromatin remodeling complex subunit Swc2 (predicted)
SPAPB1E7.08c	SPAPB1E7.08c	2.0151126	0.000435	up	membrane transporter
SPAPB1A11.03	SPAPB1A11.03	5.9188137	0.003053051	up	FMN dependent dehydrogenase
SPAC3G6.07	SPAC3G6.07	10.917849	0.0000304	up	sequence orphan
SPAC3A12.08	SPAC3A12.08	1.5740979	0.025607174	up	conserved fungal protein
SPCC1223.09	SPCC1223.09	2.9301758	0.000009	up	uricase (predicted)
SPAC1782.05	SPAC1782.05	1.6108342	0.000707	up	phosphotyrosyl phosphatase activator homolog
SPCC330.19c	SPCC330.19c	1.5302715	0.011984946	up	sequence orphan
SPAC2C4.17c	SPAC2C4.17c	1.5505787	0.011427347	up	MS ion channel protein 2
SPCC4F11.05	SPCC4F11.05	4.459856	0.003105981	up	dubious
SPAC6F6.18c	SPAC6F6.18c	1.8295747	0.03963117	up	sequence orphan
SPAC7D4.12c	SPAC7D4.12c	1.6590931	0.010221128	up	DUF1212 family protein
SPBC19G7.06	mbx1	1.867518	0.009678762	up	MADS-box transcription factor Mbx1
SPBC16E9.16c	SPBC16E9.16c	10.453439	0.005161774	up	sequence orphan
SPAC17G8.13c	mst2	1.7263985	0.007045819	up	histone acetyltransferase Mst2
SPAC26F1.04c	etr1	2.451142	0.002561124	up	enoyl-[acyl-carrier protein] reductase
SPAC212.02	SPAC212.02	14.505366	0.029525211	up	sequence orphan

SPCC4F11.04c	SPCC4F11.04c	2.0840647	0.007792226	up	mannosyltransferase complex subunit (predicted)
SPAC1751.01c	gti1	1.8339918	0.000632	up	gluconate transporter inducer Gti1
SPCC1322.08	mkp1	1.7254025	0.00632486	up	MAPK-activated protein kinase Srk1
SPBC23G7.14	SPBC23G7.14	2.318276	0.015291164	up	sequence orphan
SPCC1393.12	SPCC1393.12	1.9816903	0.000136	up	sequence orphan
SPAC29A4.12c	SPAC29A4.12c	12.390899	0.006602161	up	sequence orphan
SPCC4B3.06c	SPCC4B3.06c	2.0699005	0.00014	up	NADPH-dependent FMN reductase (predicted)
SPAC2E1P3.01	SPAC2E1P3.01	2.5302286	0.0000518	up	zinc binding dehydrogenase
SPAC222.14c	SPAC222.14c	1.5371077	0.038798593	up	GTP binding protein Sey1 (predicted)
SPAC27E2.05	cdc1	2.0343993	0.000444	up	DNA polymerase delta small subunit Cdc1
SPBC354.08c	SPBC354.08c	2.7012303	0.006466331	up	DUF221 family protein
SPCC965.07c	gst2	2.1240866	0.0000154	up	glutathione S-transferase Gst2
SPCC16C4.06c	SPCC16C4.06c	1.505234	0.004632616	up	tRNA pseudouridylate synthase (predicted)
SPBC1271.08c	SPBC1271.08c	1.8454524	0.000102	up	sequence orphan
SPBC947.05c	SPBC947.05c	2.488325	0.020821128	up	ferric-chelate reductase (predicted)
SPAC13C5.05c	SPAC13C5.05c	2.1288223	0.000177	up	N-acetylglucosamine-phosphate mutase (predicted)
SPCC31H12.02c	SPCC31H12.02c	1.7984167	0.001613344	up	membrane transporter (predicted)
SPBC725.10	SPBC725.10	2.6447399	0.001450051	up	tspO homolog
SPCC757.03c	SPCC757.03c	1.9250517	0.002314075	up	ThiJ domain protein
SPBC19C7.04c	SPBC19C7.04c	10.661568	0.001506096	up	conserved fungal protein
SPAC4C5.03	SPAC4C5.03	1.7118832	0.000337	up	CTNS domain protein (SMART)
SPAC630.04c	SPAC630.04c	1.6110173	0.003521974	up	sequence orphan
SPAPB2B4.07	SPAPB2B4.07	1.7166727	0.000227	up	ubiquitin family protein, human UBTD1 homolog
SPAC23C11.06c	SPAC23C11.06c	6.149896	0.004712509	up	hydrolase (inferred from context)
SPBC83.19c	SPBC83.19c	31.713165	0.000528	up	sequence orphan
SPBC20F10.03	SPBC20F10.03	1.5506772	0.0000257	up	conserved eukaryotic protein
SPAC11H11.04	mam2	2.6987486	0.001745679	up	pheromone p-factor receptor (PMID 1657593)
SPAC513.03	mfm2	1.6010718	0.015294003	up	M-factor precursor Mfm2
SPCC663.03	pmd1	2.1790903	0.0000874	up	leptomycin efflux transporter Pmd1
SPCC569.03	SPCC569.03	1.8323321	0.002186999	up	DUF1773 family protein 4
SPBC16E9.08	mcp4	1.595475	0.000872	up	sequence orphan
SPBC1E8.05	SPBC1E8.05	1.5546738	0.004617981	up	conserved fungal protein
SPBC15D4.13c	SPBC15D4.13c	1.7215118	0.007702763	up	sequence orphan
SPAC8C9.03	cgs1	2.0266256	0.002320234	up	cAMP-dependent protein kinase regulatory subunit Cgs1
SPAC1952.04c	SPAC1952.04c	1.7285715	0.006594122	up	dubious
SPAC1006.01	psp3	1.5630463	0.0000264	up	serine protease Psp3 (predicted)
SPAC18B11.04	ncs1	2.198244	0.000521	up	related to neuronal calcium sensor Ncs1
SPBC1773.02c	SPBC1773.02c	1.5074565	0.000235	up	thioredoxin peroxidase
SPACUNK12.02c	cmk1	2.8002427	0.000754	up	calcium/calmodulin-dependent protein kinase Cmk1
SPAC630.10	SPAC630.10	2.00998	0.00973055	up	conserved fungal protein
SPBC215.05	gpd1	1.6583283	0.005771691	up	glycerol-3-phosphate dehydrogenase Gpd1
SPAC17G8.10c	dma1	1.8733451	0.009608292	up	mitotic spindle checkpoint protein Dma1
SPBC16C6.06	pep1	1.5731393	0.005438517	up	sorting receptor for CPY
SPCC338.02	SPCC338.02	3.1724358	0.013589559	up	sequence orphan
SPBC1773.06c	SPBC1773.06c	2.1099517	0.009149103	up	alcohol dehydrogenase (predicted)
SPBC216.02	mcp5	2.1696784	0.012742977	up	cortical anchoring factor for dynein Mcp5/Num1
SPCC1739.10	SPCC1739.10	1.9216182	0.009284104	up	conserved fungal protein

SPCC4B3.10c	ipk1	1.7235159	0.003617506	up	inositol 1,3,4,5,6-pentakisphosphate (IP5) kinase (PMID 10960485)
SPBC12C2.12c	glo1	1.6186016	0.00271552	up	glyoxalase I (PMID 15042280)
SPAC458.04c	SPAC458.04c	2.1350937	0.000643	up	sequence orphan
SPAC23C11.04c	pnk1	1.6689163	0.030937605	up	DNA kinase/phosphatase Pnk1
SPCC188.13c	dcr1	2.0108964	0.028431546	up	dicer
SPCC1919.06c	wtf9	1.5030236	0.026305487	up	wtf element, Wtf2, pseudo
SPAC6G9.06c	pcp1	1.5440536	0.003708609	up	pericentrin Pcp1
SPAC167.06c	SPAC167.06c	2.2246644	0.002981554	up	sequence orphan
SPAC23D3.13c	SPAC23D3.13c	1.5585387	0.027861925	up	guanyl-nucleotide exchange factor
SPBC1861.01c	cnp3	1.5878643	0.000288	up	CENP-C
SPAC56F8.15	SPAC56F8.15	1.7727185	0.014273056	up	dubious
SPAC688.04c	gst3	2.2566175	0.001451801	up	glutathione S-transferase (PMID 12151111)
SPAC18G6.01c	SPAC18G6.01c	1.5474567	0.002281191	up	conserved fungal protein
SPBC24C6.06	gpa1	2.1290994	0.021196371	up	G-protein alpha subunit (PMID 1905818)
SPAC19B12.08	SPAC19B12.08	1.8426725	0.018612519	up	peptidase family C54
SPCPB16A4.06c	SPCPB16A4.06c	4.8622074	0.004509932	up	sequence orphan
SPAC4D7.02c	SPAC4D7.02c	1.975193	0.00112891	up	glycerophosphoryl diester phosphodiesterase (predicted)
SPAC2F3.05c	SPAC2F3.05c	2.6570551	0.000358	up	xylose and arabinose reductase (predicted)
SPBPB21E7.08		1.5668668	0.008343781	up	
SPCC4F11.02	ptc1	1.7392954	0.016378183	up	protein phosphatase 2C Ptc1
SPAC227.06	SPAC227.06	1.5518359	0.000203	up	Rab GTPase binding (predicted)
SPAC3H1.07	SPAC3H1.07	1.7207075	0.006276161	up	arginase (predicted)
SPAPB24D3.07c	SPAPB24D3.07c	2.0119555	0.000362	up	sequence orphan
SPAC19G12.09	SPAC19G12.09	1.9788798	0.002652668	up	NADH/NADPH dependent indole-3-acetaldehyde reductase AKR3C2
SPCC191.11	inv1	2.1872916	0.003411172	up	beta-fructofuranosidase
SPAC144.10c	gwt1	1.8264731	0.00255354	up	pig-W
SPAC222.15	meu13	1.9270443	0.003856817	up	Tat binding protein 1(TBP-1)-interacting protein (TBPIP) homolog (predicted)
SPAC1296.03c	sxa2	1.8423723	0.001076878	up	serine carboxypeptidase Sxa2
SPBC1198.14c	fbp1	3.5787213	0.0000536	up	fructose-1,6-bisphosphatase Fbp1 (PMID 2157626)
SPCC417.03	SPCC417.03	1.6020464	0.002341674	up	sequence orphan
SPBC106.02c	srx1	2.565769	0.00457568	up	sulphiredoxin (PMID 15824112)
SPCP31B10.06	SPCP31B10.06	1.7276497	0.001106975	up	C2 domain protein Tcb3 (predicted)
SPCC4G3.19	alp16	1.541906	0.003949614	up	gamma tubulin complex ubunit Alp16 (PMID 12134075)
SPAC5H10.02c	SPAC5H10.02c	2.847376	0.000508	up	ThiJ domain protein
SPBC428.13c	mob1	2.5308626	0.009085395	up	protein kinase regulator Mob1
SPCC1442.11c	SPCC1442.11c	2.455865	0.010917816	up	sequence orphan
SPAC16A10.01	SPAC16A10.01	3.3804839	0.006029366	up	DUF1212 family protein
SPCC584.03c	SPCC584.03c	1.6690333	0.040389407	up	RanGTP-binding protein (predicted)
SPACUNK4.17	SPACUNK4.17	5.380624	0.003651407	up	NAD binding dehydrogenase family protein
SPAC22A12.02c	SPAC22A12.02c	1.6899403	0.03765369	up	sequence orphan
SPAC3A11.10c	SPAC3A11.10c	2.332961	0.000461	up	dipeptidyl aminopeptidase (predicted)
SPAC3G9.11c	SPAC3G9.11c	2.2946577	0.011498256	up	pyruvate decarboxylase (predicted)
SPAPB1A10.08	SPAPB1A10.08	2.4263444	0.0000243	up	sequence orphan
SPBC409.09c	cnl1	2.3454382	0.024274658	up	kinetochore protein Mis13
SPCC663.08c	SPCC663.08c	1.6852895	0.005254492	up	short chain dehydrogenase (predicted)
SPBC19F8.03c	SPBC19F8.03c	1.719317	0.008320578	up	clathrin binding protein
SPBC15C4.06c	SPBC15C4.06c	3.3438506	0.000392	up	ubiquitin-protein ligase E3 (predicted)

SPBP4H10.10	SPBP4H10.10	2.4642985	0.011192657	up	rhomboid family protease
SPBC1709.01	chs2	1.5066675	0.011687819	up	chitin synthase homolog Chs2
SPCC663.07c		1.7473812	0.025764666	up	
SPCC330.04c	SPCC330.04c	1.7532583	0.000572	up	DUF1773 family protein 3
SPAC2G11.05c	SPAC2G11.05c	1.7487054	0.029232979	up	BRO1 domain protein
SPAC27D7.09c	SPAC27D7.09c	2.6088765	0.00000104	up	S. pombe specific But2 family protein
SPAC4G8.13c	prz1	2.8191762	0.000323	up	transcription factor Prz1 (PMID 12637524)
SPCC24B10.02c	SPCC24B10.02c	1.6093378	0.018540705	up	NAD/NADH kinase
SPAC6G10.03c	SPAC6G10.03c	1.831881	0.012753821	up	abhydrolase family protein, unknown biological role
SPBPB21E7.04c	SPBPB21E7.04c	27.035408	0.000161	up	S-adenosylmethionine-dependent methyltransferase (predicted)
SPCC594.04c	SPCC594.04c	1.6145649	0.004036889	up	steroid oxidoreductase superfamily protein
SPCC757.12	SPCC757.12	1.6396539	0.0030247	up	alpha-amylase homolog (predicted)
SPAC26F1.14c	aif1	3.6185567	0.022425003	up	apoptosis-inducing factor homolog Aif1
SPCC1906.04	wtf20	2.951344	0.016309492	up	wtf element Wtf20
SPAC4G9.07	SPAC4G9.07	2.8299327	0.008628846	up	S. pombe specific UPF0300 family protein 2
SPAC4H3.08	SPAC4H3.08	8.13816	0.001439643	up	short chain dehydrogenase (predicted)
SPBC1778.04	spo6	2.930875	0.0000711	up	Spo4-Spo6 kinase complex regulatory subunit Spo6
SPAC1F7.09c	SPAC1F7.09c	1.596559	0.01650246	up	allantoicase (predicted)
SPBC1604.01	SPBC1604.01	1.7197716	0.00430519	up	sulfatase modifying factor 1 related
SPAC26H5.08c	bgl2	2.7918236	0.00000172	up	glucan 1,3-beta-glucosidase Bgl2
SPCC794.15	SPCC794.15	1.5097317	0.02778388	up	sequence orphan
SPAC2E12.03c	SPAC2E12.03c	1.5167774	0.001674431	up	G-protein coupled receptor (predicted)
SPAC25B8.10	SPAC25B8.10	1.9828775	0.001942524	up	trans-aconitate 3-methyltransferase (predicted)
SPAC1B3.20	SPAC1B3.20	1.655451	0.012248222	up	sequence orphan
SPAC821.04c	cid13	1.633454	0.014600432	up	poly(A) polymerase Cid13 (PMID 12062100)
SPCC11E10.09c	SPCC11E10.09c	1.773389	0.000307	up	alpha-amylase homolog (predicted)
SPCC18B5.02c		3.235326	0.021791358	up	
SPAC18B11.03c	SPAC18B11.03c	3.656801	0.000589	up	N-acetyltransferase (predicted)
SPBP8B7.01c	SPBP8B7.01c	2.602529	0.047704566	up	sequence orphan
SPAC18B11.09c	SPAC18B11.09c	1.5641544	0.017560823	up	N-acetyltransferase (predicted)
SPBC1711.02	matmc	1.5021496	0.0000574	up	mating-type m-specific polypeptide mc
SPCC645.02	SPCC645.02	1.5216988	0.018500974	up	conserved protein (fungal and plant)
SPAPB2B4.04c	SPAPB2B4.04c	2.301566	0.000038	up	P-type ATPase, calcium transporting Pmc1 (PMID 12707717)
SPAC17A5.09c	SPAC17A5.09c	2.2102783	0.038589552	up	protein phosphatase regulatory subunit Glc9
SPAC20G4.02c	fus1	1.8941953	0.008969041	up	formin Fus1
SPAPB24D3.04c	mag1	1.5833274	0.008615806	up	DNA-3-methyladenine glycosylase Mag1
SPAC27D7.09c	PAC27D7.09c /// SPAC27D7.1(	2.2041764	0.000173	up	S. pombe specific But2 family protein /// S. pombe specific But2 family protein
SPAC15A10.10	mde6	4.9109735	0.013552912	up	Muskelin homolog
SPCC1739.08c	SPCC1739.08c	5.3278174	0.009926332	up	short chain dehydrogenase
SPAC15A10.05c	SPAC15A10.05c	1.8786193	0.009387789	up	YjeF family protein
SPBC3H7.08c	SPBC3H7.08c	1.895025	0.000774	up	conserved fungal protein
SPBC660.05	SPBC660.05	8.059851	0.021464122	up	conserved fungal protein
SPBC1711.17	prp16	1.7895286	0.011641695	up	ATP-dependent RNA helicase Prp16
SPBP4G3.03	SPBP4G3.03	8.151803	0.000166	up	PI31 proteasome regulator related
SPCC1450.08c	wtf16	2.14103	0.002338983	up	wtf element Wtf16
SPAC31G5.09c	spk1	1.6907347	0.010428346	up	MAP kinase Spk1
SPCC1259.14c	meu27	4.24529	0.000257	up	S. pombe specific UPF0300 family protein 5

SPAC6B12.08	SPAC6B12.08	1.6810993	0.018557144	up	DNAJ domain protein Jjj family
SPCC162.10	ppk33	1.5757699	0.004743667	up	serine/threonine protein kinase Ppk33 (predicted)
SPBC29A3.08	pof4	1.5184898	0.03463023	up	elongin-A, F-box protein Pof4
SPCC965.14c	SPCC965.14c	1.7895002	0.0000197	up	cytosine deaminase (predicted)
SPCC1906.04	wtf20	2.142429	0.001699505	up	wtf element Wtf20
SPCC330.03c	SPCC330.03c	1.6737986	0.0000888	up	NADPH-hemoprotein reductase
SPCC1906.04	wtf20	1.856457	0.003732963	up	wtf element Wtf20
SPBC83.09c	SPBC83.09c	1.650007	0.02232206	up	GYF domain
SPAC1B3.06c	SPAC1B3.06c	2.2607741	0.0000183	up	UbiE family methyltransferase (predicted)
SPAC664.13	SPAC664.13	1.6038228	0.016375553	up	sequence orphan
SPBC1198.07c	SPBC1198.07c	1.5369924	0.014546962	up	mannan endo-1,6-alpha-mannosidase (predicted)
SPAC14C4.07	SPAC14C4.07	1.6724548	0.005608005	up	membrane transporter
SPCC1739.15	wtf21	1.3466578	0.020875992	up	wtf element Wtf21
SPAC22G7.11c	SPAC22G7.11c	2.130058	0.002285681	up	conserved fungal protein
SPBC557.05	SPBC557.05	1.6463442	0.010693104	up	arrestin
SPAC19D5.03	cid1	1.7469833	0.03916173	up	poly(A) polymerase Cid1
SPAC13D6.04c	btb3	1.6736825	0.011442144	up	BTB/POZ domain protein Btb3
SPCC417.05c	cfh2	3.01746	0.000388	up	chitin synthase regulatory factor (putative) Chr2 (PMID 15449309)
SPAPJ691.02	SPAPJ691.02	5.4534574	0.006538227	up	yippee-like protein
SPCC622.05	SPCC622.05	2.0819917	0.038559888	up	dubious
SPCC61.05	SPCC61.05	1.5407017	0.012501651	up	S. pombe specific multicopy membrane protein family 1
SPAC31G5.18c	SPAC31G5.18c	1.8258116	0.011011207	up	ubiquitin family, human C1ORF55 related
SPAC56F8.12	SPAC56F8.12	1.6999134	0.036387015	up	conserved fungal protein
SPAPB17E12.09	SPAPB17E12.09	2.1061442	0.013677208	up	sequence orphan
SPAC212.06c	tlh1 /// tlh2	3.6738126	0.018553376	up	RecQ type DNA helicase /// RecQ type DNA helicase Tlh1
SPBC19C7.08c	SPBC19C7.08c	1.5568119	0.00140432	up	leucine carboxyl methyltransferase
SPBC725.03	SPBC725.03	3.3421059	0.0000467	up	conserved fungal protein
SPAC6B12.03c	SPAC6B12.03c	6.9190073	0.0000311	up	HbrB family protein
SPAC26F1.11	SPAC26F1.11	4.696152	0.000403	up	sequence orphan
SPBC365.13c	caf1	1.5100296	0.004909408	up	Ran GTPase binding protein Hba1
SPAC26F1.10c	pyp1	1.5191364	0.002312676	up	tyrosine phosphatase Pyp1
SPAC29B12.11c	SPAC29B12.11c	1.7582095	0.001466533	up	human WW domain binding protein-2 ortholog
SPAC22H10.11c	SPAC22H10.11c	1.7852927	0.032521762	up	protein coding
SPAC2E1P3.04	SPAC2E1P3.04	1.5704805	0.003378623	up	copper amine oxidase (predicted)
SPAC1D4.03c	aut12	2.3168764	0.03187874	up	autophagy associated protein Aut12
SPAC688.03c	SPAC688.03c	1.5094318	0.000993	up	human AMMECR1 homolog
SPBC32H8.07	git5	1.592312	0.022363827	up	heterotrimeric G protein beta subunit Git5
SPCC74.09	SPCC74.09	1.8249308	0.029053338	up	RNA-binding protein
SPCC970.11c	wtf9	2.8891046	0.021807918	up	wtf element, Wtf2, pseudo
SPBC25B2.08	SPBC25B2.08	1.5599529	0.000098	up	sequence orphan
SPCC576.01c	SPCPB1C11.04c	1.9942579	0.003005279	up	sulfonate dioxygenase (predicted)
SPAC22E12.09c	krp	1.6076854	0.012289964	up	kexin
SPAPB18E9.04c	SPAPB18E9.04c	2.2015007	0.000882	up	sequence orphan
SPBC800.14c	SPBC800.14c	1.9185404	0.008491046	up	DUF1772 family protein
SPCC338.18	SPCC338.18	1.7915833	0.007033632	up	sequence orphan
SPBC1347.03	meu14	1.7916343	0.010787684	up	sporulation protein Meu14
SPBC839.02	SPBC839.02	1.6163605	0.001478504	up	arrestin Aly1 related

SPAC17A2.05	SPAC17A2.05	2.2390985	0.0000284	up	fumerate reductase
SPBP18G5.03	SPBP18G5.03	1.5285295	0.011092929	up	sequence orphan
SPAC26H5.09c	SPAC26H5.09c	1.6892668	0.005209245	up	GFO/IDH/MocA family oxidoreductase
SPAC4G9.02	SPAC4G9.02	1.8164746	0.000129	up	ribonuclease H2 complex subunit
SPBC119.03	SPBC119.03	3.938327	0.0000185	up	S-adenosylmethionine-dependent methyltransferase (predicted)
SPCC1442.02	SPCC1442.02	1.5618224	0.002182023	up	DUF1760 family protein
SPAC22G7.07c	SPAC22G7.07c	1.6825167	0.02998393	up	mRNA (N6-adenosine)-methyltransferase (predicted)
SPBC713.12	erg1	1.5631621	0.004430475	up	squalene monooxygenase Erg1 (predicted)
SPBC365.12c	ish1	2.6466863	0.000332	up	LEA domain protein
SPAC1565.04c	ste4	1.7865134	0.00123483	up	adaptor protein Ste4
SPAC2G11.13	atg22	2.2368264	0.0000584	up	autophagy associated protein Atg22 (predicted)
SPCC18B5.01c	SPCPJ732.04c	1.5792325	0.003009974	up	brefeldin A efflux transporter Bfr1
SPCC132.04c	SPCC132.04c	2.0881515	0.001373293	up	NAD-dependent glutamate dehydrogenase (predicted)
SPBC215.11c	SPBC215.11c	2.9854996	0.001933469	up	aldo/keto reductase, unknown biological role
SPBCPT2R1.02	SPAC212.02 /// SPBCPT2R1.02	8.047846	0.000783	up	sequence orphan /// sequence orphan
SPAC22H10.12c	gdi1	2.092262	0.033718843	up	GDP dissociation inhibitor Gdi1 (predicted)
SPAC22G7.08	ppk8	1.6073486	0.009759226	up	serine/threonine protein kinase Ppk8 (predicted)
SPBC21C3.19	SPBC21C3.19	2.5813024	0.013469043	up	DUF1960 family protein
SPAC1834.09	SPAC1834.09	1.642204	0.001194319	up	conserved fungal protein
SPAC6B12.06c	SPAC6B12.06c	2.104702	0.029857343	up	conserved fungal protein
SPAC513.02	SPAC513.02	2.1566675	0.001612547	up	phosphoglycerate mutase family
SPAC222.11	hem13	1.568304	0.00489947	up	coproporphyrinogen III oxidase (predicted)
SPAC4H3.03c	SPAC4H3.03c	4.3192654	0.0000691	up	glucan 1,4-alpha-glucosidase (predicted)
SPAC869.02c	SPAC869.02c	2.4773092	0.00000472	up	nitric oxide dioxygenase (predicted)
SPBC23G7.10c	SPBC23G7.10c	2.2497876	0.000521	up	NADH-dependent flavin oxidoreductase (predicted)
SPAPB17E12.07c	sen2	1.929908	0.038710117	up	tRNA-splicing endonuclease subunit Sen2
SPAC20H4.11c	rho5	1.6547481	0.000399	up	Rho family GTPase Rho5
SPAC5D6.07c	SPAC5D6.07c	1.5351312	0.028311785	up	PXA domain protein
SPBC887.15c	SPBC887.15c	1.6402295	0.002555209	up	sphingosine hydroxylase (predicted)
SPAC14C4.01c	SPAC14C4.01c	1.7171748	0.00845649	up	DUF1770 family protein
SPAC2F3.16	SPAC2F3.16	1.5690231	0.012998533	up	ubiquitin-protein ligase E3 (predicted)
YLR432W	IMD3	2.7619073	0.019508278	up	genase, catalyzes the first step of GMP biosynthesis, member of a four-gene family in S. cerevisiae, constitutively expressed
SPBC660.06	SPBC660.06	3.288425	0.0000022	up	conserved fungal protein
SPAC23H3.15c	SPAC23H3.15c	9.259536	0.008475055	up	sequence orphan
SPAC17C9.16c	mfs1	2.3261085	0.049438644	up	MFS family transmembrane transporter Mfs1
SPCC1739.15	wtf19 /// wtf21 /// wtf23	1.6942003	0.017901639	up	wtf element Wtf19 /// wtf element Wtf21 /// wtf element Wtf23
SPAC15E1.02c	SPAC15E1.02c	5.7806773	0.0000235	up	DUF1761 family protein
SPAC227.04	SPAC227.04	1.6309961	0.000849	up	autophagy C terminal domain family protein
SPCC338.12	SPCC338.12	1.8627478	0.001490923	up	protease inhibitor (predicted)
SPAC19D5.01	pyp2	2.291514	0.013946873	up	tyrosine phosphatase Pyp2
SPAC22A12.17c	SPAC22A12.17c	3.7031057	0.002471715	up	short chain dehydrogenase (predicted)
SPBP23A10.12	SPBP23A10.12	2.0817494	0.001752352	up	FRG1 family protein
SPAC3C7.13c	SPAC3C7.13c	1.836798	0.001487057	up	glucose-6-phosphate 1-dehydrogenase (predicted)
SPBC428.10	SPBC428.10	1.8342415	0.001496578	up	sequence orphan
SPAPB24D3.10c	agl	2.3184798	0.0000493	up	alpha-glucosidase Agl1
SPAC24B11.14	SPAC24B11.14	1.7907101	0.018258775	up	sequence orphan
SPBC947.13	rba50	1.5837272	0.002778101	up	RNA polymerase II associated protein (predicted)

SPBC1685.13	SPBC1685.13	2.3894129	0.006622838	up	non classical export pathway protein (predicted)
SPCC10H11.02	SPCP31B10.01	1.6095335	0.003754997	up	DNAJ domain protein Cwf23
SPAC29E6.05c	SPAC29E6.05c	2.9262872	0.03892624	up	peptide methionine sulfoxide reductase (predicted)
SPMIT.07		1.6651134	0.020733329	up	
SPCC757.07c	cta1	2.40218	0.004007064	up	catalase
SPAC23H3.04	SPAC23H3.04	1.9948546	0.001163937	up	conserved fungal protein
SPBC1685.05	SPBC1685.05	1.8018348	0.00112097	up	serine protease (predicted)
SPAC750.07c	SPAC212.08c /// SPAC750.07c	2.3267713	0.031590164	up	GPI anchored protein (predicted) /// S. pombe specific GPI anchored protein family 1
SPBC17D11.08	SPBC17D11.08	1.778722	0.000188	up	WD repeat protein, human WDR68 family
SPBC3H7.13	SPBC3H7.13	1.6592921	0.000447	up	FHA domain protein Far10 (predicted)
SPAC4A8.04	isp6	1.5815747	0.002249675	up	vacuolar serine protease Isp6
SPBC359.02	alr2	1.6566267	0.000577	up	alanine racemase Alr2 (PMID 11244061)
SPAC31G5.02	SPAC31G5.02	1.5344144	0.000365	up	conserved fungal protein
SPAC513.06c	SPAC513.06c	2.5305314	0.04562532	up	dihydrodiol dehydrogenase (predicted)
SPAC2F7.16c	SPAC2F7.16c	1.7226821	0.003508127	up	phospholipase D (predicted)
SPCC1281.04	SPCC1281.04	4.143537	0.000155	up	pyridoxal reductase (predicted)
SPAC630.05	gyp7	2.213945	0.004635263	up	GTPase activating protein Gyp7 (predicted)
SPAC824.02	SPAC824.02	2.9066255	0.003668563	up	GPI inositol deacylase
SPCC1235.13	ght6	2.669563	0.018709995	up	hexose transporter Ght6 (PMID 10735857)
SPCC417.06c	mug27	20.797619	0.03672741	up	serine/threonine protein kinase Ppk35
SPAC637.03	SPAC637.03	7.2532296	0.0000871	up	conserved fungal protein
SPBC1604.18c	SPBC1604.18c	1.9246147	0.005688468	up	vacuolar sorting protein (predicted)
SPCP20C8.02c	SPCP20C8.02c	1.5102776	0.03990427	up	S. pombe specific UPF0321 family protein 1
SPCC13B11.04c	SPCC13B11.04c	1.5971475	0.025446063	up	glutathione-dependent formaldehyde dehydrogenase
SPAC343.07	mug28	1.8800638	0.024706265	up	RNA-binding protein Mug28
SPCC1450.09c	SPCC1450.09c	1.9924487	0.001390275	up	phospholipase (predicted)
YLL025W	PAU17	1.5471365	0.005208899	up	Putative protein of unknown function; YLL025W is not an essential gene
SPAP8A3.12c	SPAP8A3.12c	1.5206625	0.00308898	up	tripeptidylpeptidase (predicted)
SPAC15A10.09c	SPAC15A10.09c	1.7984215	0.001975183	up	conserved fungal protein
SPCC4B3.02c	SPCC4B3.02c	2.1409829	0.005017106	up	Golgi transport protein Got1 (predicted)
SPAC2F3.01	SPAC2F3.01	2.6618264	0.00000418	up	mannosyltransferase complex subunit
SPAC2F3.13c	SPAC2F3.13c	1.5176866	0.020226946	up	queuine tRNA-ribosyltransferase (predicted)
SPAC23G3.03	sib2	1.9714344	0.0000136	up	ornithine N5 monooxygenase (predicted)
SPBPB2B2.18	SPBPB2B2.18	12.480143	0.009551786	up	dubious
SPBC56F2.06	SPBC56F2.06	11.039784	0.001614729	up	sequence orphan
SPBC11C11.03	ndc10	1.6067011	0.003386969	up	spindle pole body protein Ndc80
SPBC15D4.02	SPBC15D4.02	1.6000075	0.002351706	up	transcription factor
SPCC622.21	wtf12	1.7413563	0.04204796	up	wtf element Wtf12
SPAC1B3.15c	SPAC1B3.15c	1.7322783	0.000586	up	membrane transporter
SPAC25H1.03	mug66	1.674135	0.000159	up	meiotically upregulated gene Mug66
SPAC25G10.04c	rec10	3.167331	0.0000788	up	meiotic recombination protein Rec10 (PMID 7586030)
SPBC14C8.11c	SPBC14C8.11c	2.3036435	0.019377537	up	sequence orphan
SPAC22F8.05	SPAC22F8.05	3.3163507	0.00062	up	alpha,alpha-trehalose-phosphate synthase (predicted)
SPAC1527.02	sft2	1.7815956	0.021773152	up	Golgi transport protein Sft2 (predicted)
SPAC1687.07	SPAC1687.07	1.796752	0.011017026	up	conserved fungal protein
SPAC25B8.09	SPAC25B8.09	2.4038749	0.004290735	up	trans-aconitate 3-methyltransferase (predicted)
SPAC27F1.05c	SPAC27F1.05c	3.084917	0.00350787	up	4-aminobutyrate transaminase

SPAC3C7.05c	SPAC3C7.05c	1.696237	0.007037418	up	alpha-1,6-mannanase (predicted)
SPBC119.04	mei3	2.308515	0.001273673	up	meiosis inducing protein Mei3 (PMID 3034608)
SPBC3D6.03c	SPBC3D6.03c	1.6423148	0.005829041	up	tRNA endonuclease (predicted)
SPAC27E2.04c	SPAC27E2.04c	2.3073423	0.000021	up	sequence orphan
SPCC1322.10	SPCC1322.10	1.8366226	0.0001	up	conserved fungal protein
SPBC19C7.12c	SPBC19C7.12c	2.2605531	0.000426	up	alpha-1,2-mannosyltransferase
SPCC285.07c	wtf13 /// wtf18	2.548128	0.030430038	up	wtf element Wtf13 /// wtf element Wtf18
SPCC191.01	SPCC191.01	2.0401876	0.001829994	up	sequence orphan
SPBC365.20c	SPBC365.20c	1.9100267	0.015610484	up	nicotinamidase (predicted)
SPBC28F2.07	dds20	1.6144966	0.001121465	up	Swi five-dependent recombination repair protein Sfr1
SPAC139.05	SPAC139.05	2.571139	0.000764	up	succinate-semialdehyde dehydrogenase (predicted)
SPAC8E11.03c	dmc1	2.4340882	0.0102108	up	RecA family ATPase Dmc1
SPCC70.10	SPCC70.10	2.2375975	0.001964835	up	sequence orphan
SPCC16C4.20c	SPCC16C4.20c	2.3751605	0.038968142	up	sequence orphan
SPAC186.01	SPAC186.01	1.9711014	0.000963	up	DIPSY family
SPAC23C11.10	SPAC23C11.10	1.5653806	0.001869671	up	conserved eukaryotic protein
SPCC584.16c	SPCC584.16c	3.69167	0.000319	up	sequence orphan
SPAPB18E9.02c	ppk18	1.8401772	0.025948564	up	serine/threonine protein kinase Ppk18 (predicted)
SPBC1271.09	SPBC1271.09	2.7849596	0.008905653	up	glycerophosphodiester transporter
SPCC622.11	SPCC622.11	1.5281093	0.008218526	up	LMBR1-like membrane protein
SPBC146.06c	SPBC146.06c	1.6372224	0.011121484	up	human MTMR15 homolog
SPAC1F7.12	yak3	1.606072	0.000255	up	aldose reductase YakC
SPAC6F12.15c	cut9	1.5529139	0.03516838	up	anaphase-promoting complex subunit Cut9
SPBC1604.17c	SPBC1604.17c	1.6569613	0.00230373	up	conserved fungal protein
SPAC13G7.05	SPAC13G7.05	1.5727266	0.001707126	up	acyl-coA-sterol acyltransferase (predicted)
SPBC1198.01	SPBC1198.01	1.6042204	0.000663	up	glutathione-dependent formaldehyde dehydrogenase (predicted)
SPCC737.04	SPCC737.04	33.826565	0.0000387	up	S. pombe specific UPF0300 family protein 6
SPCC622.15c	SPCC622.15c	1.6484939	0.031197673	up	sequence orphan
SPBC36B7.02	SPBC36B7.02	1.9204221	0.000303	up	Svf1 family protein Svf2
SPCC4G3.03	SPCC4G3.03	2.0376415	0.020946631	up	WD repeat protein
SPBC405.04c	ypt7	2.5881436	0.028923087	up	GTPase Ypt7
SPAPB24D3.08c	SPAPB24D3.08c	2.307501	0.000575	up	NADP-dependent oxidoreductase (predicted)
SPCC417.10	SPCC417.10	1.5421145	0.010161636	up	membrane transporter
SPBC32C12.02	aff1	2.6494434	0.0000228	up	transcription factor Ste11
SPAC19E9.03	pas1	2.1556098	0.00000425	up	cyclin Pas1
SPBC1105.14	rsv2	1.9225566	0.000297	up	transcription factor Rsv2
SPAC19A8.05c	vps27	1.5682013	0.003637204	up	sorting receptor for ubiquitinated membrane proteins (ISS) (PMID 12055639)
SPCC306.02c	SPCC306.02c	1.7517431	0.003597254	up	Rab GTPase binding (predicted)
SPAC23A1.14c	SPAC23A1.14c	1.811745	0.002710653	up	cystathionine gamma-synthase (predicted)
SPBC1683.08	ght4	1.6394479	0.020391064	up	hexose transporter Ght4 (PMID 10735857)
SPCC16A11.15c	SPCC16A11.15c	2.4360037	0.000288	up	sequence orphan
SPBP22H7.04	SPBP22H7.04	1.579338	0.006920552	up	sequence orphan
SPAC11E3.06	map1	3.6855118	0.00402006	up	MADS-box transcription factor Map1
SPAC1D4.11c	kic1	1.7417701	0.002623418	up	dual specificity protein kinase Lkh1
SPBC36.04	cys11	1.6469668	0.04692993	up	cysteine synthase (PMID 14981292)
SPAC11H11.05c	fta6	1.6722054	0.027252328	up	Sim4 and Mal2 associated (4 and 2 associated) protein 6 (PMID 16079914)
SPBC19F8.05	SPBC19F8.05	1.7326683	0.003049546	up	sequence orphan

SPAC17G6.02c	SPAC17G6.02c	2.6900718	0.0021682	up	RTA1-like protein
SPCPB1C11.02	SPCPB1C11.02	5.4845753	0.000527	up	amino acid permease, unknown 16
SPBC119.05c	SPBC119.05c	3.2558546	0.011684343	up	Wiskott-Aldrich syndrome homolog binding protein Lsb1 (predicted)
SPAC23G3.07c	snf30	2.119964	0.000779	up	SWI/SNF complex subunit Snf30
SPAC29A4.17c	SPAC29A4.17c	1.8231504	0.008716346	up	FUN14 family protein

Supplemental Table 1B: The gene list that down-regulated by resveratrol

Transcript_ID(Array_Design)	Gene_Symbol	Relative Ratio	p-value	Regulation	Gene_Title
SPBC1105.11c	h3.3	0.651568394	0.0000431	down	histone H3 h3.3
SPAC3H5.06c	pol1	0.649138752	0.005009082	down	DNA polymerase alpha catalytic subunit (PMID 2034212)
SPBC1683.07	mal1	0.560540065	0.004047691	down	alpha-glucosidase Mal1 (predicted)
SPBC16D10.06	SPBC16D10.06	0.66322352	0.000127	down	ZIP zinc transporter 2
SPBC1105.12	h4.3	0.808704507	0.047392126	down	histone H4 h4.3
SPBPB21E7.07	aes1	0.382078822	0.0000172	down	enhancer of RNA-mediated gene silencing (PMID 12034844)
SPAC186.03	SPAC186.03 /// SPBPB21E7.09	0.539298958	0.004008098	down	L-asparaginase /// L-asparaginase (predicted)
SPCC290.04	ams2	0.504012646	0.014569213	down	cell cycle regulated GATA-type transcription factor Ams2
SPBC1105.16c	rpr2	0.662264725	0.009201759	down	RNase P subunit Rpr2 (predicted)
SPCC1672.02c	sap1	0.636056814	0.000975	down	switch-activating protein Sap1
SPCC1672.01	SPCC1672.01	0.659750285	0.000779	down	histidinol-phosphatase (predicted)
SPAC922.06	SPAC922.06	0.602926848	0.000594	down	short chain dehydrogenase
SPAC977.09c	SPAC977.09c /// SPBC1348.10c	0.339840717	0.000114	down	phospholipase (predicted) /// phospholipase (predicted)
SPAC977.07c	SPAC977.07c /// SPBC1348.08c	0.41525887	0.018526426	down	glycoprotein (predicted) /// glycoprotein (predicted)
SPAC2E1P5.03	SPAC2E1P5.03	0.60809618	0.000773	down	DNAJ domain protein Erj5
SPBC428.18	cdt1	0.461225765	0.00017	down	replication licensing factor Cdt1
SPBPB2B2.01	SPBPB2B2.01	0.208224493	0.006592532	down	amino acid permease, unknown 12
SPAC1B3.16c	vht1	0.61074737	0.000835	down	vitamin H transporter Vth1
SPBPB10D8.01	SPBPB10D8.01	0.208994732	0.000403	down	cysteine transporter (predicted)
SPAC23H3.13c	git8	0.608452044	0.001696953	down	heterotrimeric G protein alpha-2 subunit Gpa2 (PMID 1340462)
SPBC359.05	abc3	0.664927659	0.001624302	down	ABC transporter Abc3
SPAC977.16c	dak2	0.650110772	0.000879	down	dihydroxyacetone kinase Dak2 (PMID 9804990)
SPAC644.05c	SPAC644.05c	0.648854571	0.023150371	down	deoxyuridine 5'-triphosphate nucleotidohydrolase (predicted)
SPBC1683.02	SPBC1683.02	0.58463952	0.003369843	down	adenine deaminase (predicted)
SPAC9.10	SPAC9.10	0.640153637	0.016317533	down	amino acid permease, unknown 2
SPAC926.05c	SPAC926.05c	0.596342998	0.000162	down	diphthamide biosynthesis protein Dph4 (predicted)
SPAC977.15	SPAC977.15	0.57590271	0.000584	down	dienelactone hydrolase family
SPBC31F10.17c	SPBC31F10.17c	0.620032301	0.001387483	down	sequence orphan
SPBC14C8.07c	cdc18	0.62908528	0.000368	down	MCM loader
SPBC18H10.21c	SPBC18H10.21c	0.665611451	0.021233011	down	dubious
SPAC1F7.05	cdc22	0.46987374	0.000809	down	ribonucleoside reductase large subunit Cdc22
SPCPB1C11.03	SPCPB1C11.03	0.563514414	0.000358	down	cysteine transporter (predicted)
SPBC409.08	SPBC409.08	0.571531708	0.000368	down	spermine family transporter (predicted)
SPAC186.05c	SPAC186.05c	0.634958491	0.006112753	down	human TMEM165 homolog
SPAC17H9.19c	cdt2	0.635201576	0.000209	down	WD repeat protein Cdt2
SPBC725.17c	rrn11	0.640100736	0.003723062	down	RNA polymerase I transcription factor subunit Rrn11 (predicted)
SPAC1002.17c	SPAC1002.17c	0.633286795	0.02310152	down	uracil phosphoribosyltransferase (predicted)
SPAPB1E7.04c	SPAPB1E7.04c	0.44785852	0.00320776	down	chitinase (predicted)
SPBC25D12.03c	mcm7	0.638446766	0.012378019	down	MCM complex subunit Mcm7
SPAPYUG7.03c	mid2	0.571664718	0.0000343	down	anillin homologue Mid2
SPBC8E4.02c	SPBC8E4.02c	0.412887081	0.003508051	down	sequence orphan
SPBPB7E8.01	SPBPB7E8.01	0.373323665	0.000186	down	sequence orphan
SPBC3B8.08	SPBC3B8.08	0.659420688	0.00000439	down	Sjogren's syndrome/scleroderma autoantigen 1 family

SPAC4G8.12c	SPAC4G8.12c	0.615562314	0.002563617	down	alpha-1,2-mannosyltransferase (predicted)
SPCC18.01c	adg3	0.473084854	0.0000738	down	beta-glucosidase Adg3 (predicted)
SPCC297.04c	set7	0.660203974	0.005160559	down	histone lysine methyltransferase Set7 (predicted)
SPAC1834.12	pex7	0.610632318	0.00017	down	peroxin-7
SPAC14C4.09	agn1	0.464574499	0.000118	down	glucan endo-1,3-alpha-glucosidase Agn1
SPAC1F8.04c	SPAC1F8.04c	0.607889432	0.012680622	down	hydrolase (predicted)
SPBPB2B2.02	SPBPB2B2.02	0.605764222	0.027253663	down	esterase/lipase (predicted)
SPBC359.01	SPBPB10D8.08	0.652769679	0.002309062	down	amino acid permease, unknown 7
SPAC13G6.03	gpi7	0.664682368	0.004870514	down	GPI anchor biosynthesis protein Gpi7 (predicted)
SPCC306.11	SPCC306.11	0.614211038	0.000338	down	sequence orphan
SPBC8E4.01c	SPBP4G3.01	0.523020212	0.001010291	down	inorganic phosphate transporter (predicted)
SPBC1734.05c	spf31	0.653867686	0.005248474	down	DNAJ protein Spf31
SPBC17G9.08c	csx2	0.623805062	0.002005139	down	Arf GAP protein
SPAC821.09	eng1	0.480993702	0.034171037	down	endo-1,3-beta-glucanase Eng1
SPAC5H10.07	SPAC5H10.07	0.505890385	0.003285816	down	sequence orphan
SPBC660.14	mik1	0.545699733	0.02730544	down	mitotic inhibitor kinase Mik1
SPBC1105.12	h4.3	0.688354041	0.00071	down	histone H4 h4.3
SPAC323.07c	SPAC323.07c	0.631059034	0.004857972	down	MatE family transporter
SPCC4B3.11c	SPCC4B3.11c	0.573296108	0.000398	down	conserved eukaryotic protein
SPAC1039.01	SPAC1039.01	0.498505978	0.00019	down	amino acid permease, unknown 5
SPBCPT2R1.08c	tlh2	0.395549592	0.006932829	down	RecQ type DNA helicase Tlh1
SPAC27D7.03c	mei2	0.471075605	0.000881	down	RNA-binding protein involved in meiosis Mei2
SPBC800.11	SPBC800.11	0.538966583	0.002559118	down	inosine-uridine preferring nucleoside hydrolase (predicted)
SPAC25B8.13c	isp7	0.585677914	0.014500576	down	2-OG-Fe(II) oxygenase superfamily protein
SPAC1834.03c	h4.1	0.596582715	0.016832162	down	histone H4 h4.1
SPAC110.01	ppk1	0.516361722	0.001265533	down	serine/threonine protein kinase Ppk1 (predicted)
SPAC869.10c	SPAC869.10c	0.373500889	0.0000505	down	proline specific permease (predicted)
SPBC1271.10c	SPBC1271.10c	0.663055182	0.00096	down	membrane transporter
SPAC19G12.16c	adg2	0.578287524	0.000643	down	hypothetical protein
SPAC11D3.04c	SPAC11D3.04c	0.588681237	0.004951982	down	SnoaL
SPAC1786.02	SPAC1786.02	0.49115419	0.000408	down	phospholipase (predicted)
SPAC5H10.06c	adh4	0.500143116	0.001086892	down	alcohol dehydrogenase Adh4
SPBC3H7.07c	SPBC3H7.07c	0.663824877	0.01026455	down	phosphoserine phosphatase (predicted)
SPAC1039.02	SPAC1039.02	0.597963778	0.02379694	down	phosphoprotein phosphatase (predicted)
SPCC338.11c	rrg1	0.638950894	0.005396711	down	methyltransferase (predicted)
SPCPB1C11.01	amt1	0.479801255	0.000739	down	ammonium transporter Amt1
SPBC2G2.05	rpl1603	0.651840213	0.00011	down	60S ribosomal protein L13/L16
SPAC29B12.03	spd1	0.622201803	0.006637509	down	ribonucleotide reductase (RNR) inhibitor
SPAC1834.04	hht1	0.748084492	0.00018	down	histone H3 h3.1
SPAC24H6.11c	SPAC24H6.11c	0.628574586	0.00110616	down	sulfate transporter (predicted)
SPBC23E6.09	ssn6	0.589643954	0.004493592	down	transcriptional corepressor Ssn6
SPBPB2B2.09c	SPBPB2B2.09c	0.509480437	0.000127	down	2-dehydropantoate 2-reductase (predicted)
SPBC1773.13	SPBC1773.13	0.583630961	0.006331075	down	aromatic aminotransferase (predicted)
SPBC359.06	SPBC359.06	0.586718179	0.02643291	down	adducin
SPBC2A9.13	SPBC2A9.13	0.649712505	0.016251912	down	sequence orphan
SPBPB10D8.02c	SPBPB10D8.02c	0.240828527	0.000721	down	arylsulfatase (predicted)
SPAC1834.04	hht1	0.839225465	0.038001202	down	histone H3 h3.1

SPBC211.05	SPBC211.05	0.597167479	0.002678585	down	splicing factor 3B
SPAPJ760.03c	adg1	0.493637019	0.000692	down	sequence orphan
SPBC1773.12	SPBC1773.12	0.424382002	0.007093672	down	transcription factor (predicted)
SPBC359.04c	SPBC359.04c	0.169641126	0.00078	down	DIPSY family
SPAC29B12.10c	SPAC29B12.10c	0.599857786	0.0000229	down	OPT oligopeptide transporter family
SPAC6G10.12c	ace2	0.524955381	0.0000865	down	transcription factor Ace2
SPBC3E7.12c	cfh4	0.567043736	0.021054814	down	chitin synthase regulatory factor (putative) Chr1 (PMID 15449309)
SPBCPT2R1.08c	tlh1 /// tlh2	0.414134236	0.007776863	down	RecQ type DNA helicase /// RecQ type DNA helicase Tlh1
SPBC8E4.03	SPBC8E4.03	0.640580766	0.000786	down	agmatinase 2 (predicted)
SPBPB2B2.13	SPBPB2B2.13	0.413744304	0.00129648	down	galactokinase Gal1 (predicted)
SPBPB10D8.03		0.196721766	0.000966	down	
SPBC2A9.07c	SPBC2A9.07c	0.507884217	0.000817	down	zf-PARP-type zinc finger protein
SPBPB2B2.05	SPBPB2B2.05	0.276354002	0.000426	down	GMP synthase [glutamine-hydrolyzing] (predicted)
