# Supplementary material for: The Natural Product Resveratrol Inhibits Yeast Cell Separation by Extensively Modulating the Transcriptional Landscape and Reprogramming the Intracellular Metabolome
Source: PLoS One. 2016 Mar 7;11(3):e0150156. doi: 10.1371/journal.pone.0150156 (PMC4780762; doi:10.1371/journal.pone.0150156)
Supplement: S2 Table — The gene lists indicates the overlap between resveratrol regulated genes and Sak1/Fkh2/Sep1 transcription factor downstream binding target genes (the Chip-seq datasets were adapted from Garg A et al. [11]). (PDF) [file pone.0150156.s003.pdf]

Supplemental Table 2: The resveratrol regulated genes are direct target of Sak1/Fhk2/Sep1 transcription factors

Transcript_ID(Array_Design)	Gene_Symbol	Sak1 target	Fhk2 target	Sep1 target
SPAC15A10.09c	pun1			
SPAC15A10.10	mde6			
SPAC19G12.16c	adg2			
SPAC6G10.12c	ace2			
SPAC821.04c	cid13			
SPAC821.09	eng1			
SPAPB1E7.04c	SPAPB1E7.04c			
SPBC1E8.05	SPBC1E8.05			
SPCC306.11	SPCC306.11			
SPCC338.18	SPCC338.18			
SPCC794.15	SPCC794.15			
SPAC16A10.01	SPAC16A10.01			
SPAC22H10.12c	gdi1			
SPAC27D7.03c	mei2			
SPAC31G5.09c	spk1			
SPAC513.03	mfm2			
SPAC5H10.06c	adh4			
SPAC5H10.07	SPAC5H10.07			
SPAC644.05c	dut1			
SPAPB1A10.08	SPAPB1A10.08			
SPBC119.04	mei3			
SPBC1198.07c	SPBC1198.07c			
SPBC1683.08	ght4			
SPBC1709.01	chs2			
SPBC19C7.04c	SPBC19C7.04c			
SPBC428.18	cdt1			
SPBC660.05	SPBC660.05			
SPBC660.14	mik1			
SPBPB2B2.13	gal1			
SPCC1322.10	SPCC1322.10			
SPCC320.14	sry1			
SPCC330.04c	mug135			
SPCC663.03	pmd1			
SPCC74.09	mug24			
SPCC757.12	SPCC757.12			
SPAC1705.03c	ecm33			
SPAC19B12.02c	gas1			
SPAPB1E7.05	gde1			
SPBC1105.05	exg1			
SPCC825.03c	psy1			

Sak1 binding targets

Fhk2 binding targets

Sep1 binding targets
